# Recovery of consciousness after acute brain injury: a narrative review

**DOI:** 10.1186/s40560-024-00749-9

**Published:** 2024-09-26

**Authors:** Satoshi Egawa, Jeremy Ader, Jan Claassen

**Affiliations:** 1 Department of Neurology, Neurological Institute, Columbia University Medical Center, NewYork-Presbyterian Hospital, 177 Fort Washington Avenue, MHB 8 Center, Room 300, New York, NY 10032 USA; 2 NewYork-Presbyterian Hospital, New York, NY USA

**Keywords:** Disorders of consciousness, Coma, Mesocircuit model, Coma recovery scale-revised, Cognitive motor dissociation, MRI, EEG, Curing coma campaign

## Abstract

**Background:**

Disorders of consciousness (DoC) are frequently encountered in both, acute and chronic brain injuries. In many countries, early withdrawal of life-sustaining treatments is common practice for these patients even though the accuracy of predicting recovery is debated and delayed recovery can be seen. In this review, we will discuss theoretical concepts of consciousness and pathophysiology, explore effective strategies for management, and discuss the accurate prediction of long-term clinical outcomes. We will also address research challenges.

**Main text:**

DoC are characterized by alterations in arousal and/or content, being classified as coma, unresponsive wakefulness syndrome/vegetative state, minimally conscious state, and confusional state. Patients with willful modulation of brain activity detectable by functional MRI or EEG but not by behavioral examination is a state also known as covert consciousness or cognitive motor dissociation. This state may be as common as every 4th or 5th patient without behavioral evidence of verbal command following and has been identified as an independent predictor of long-term functional recovery. Underlying mechanisms are uncertain but intact arousal and thalamocortical projections maybe be essential. Insights into the mechanisms underlying DoC will be of major importance as these will provide a framework to conceptualize treatment approaches, including medical, mechanical, or electoral brain stimulation.

**Conclusions:**

We are beginning to gain insights into the underlying mechanisms of DoC, identifying novel advanced prognostication tools to improve the accuracy of recovery predictions, and are starting to conceptualize targeted treatments to support the recovery of DoC patients. It is essential to determine how these advancements can be implemented and benefit DoC patients across a range of clinical settings and global societal systems. The Curing Coma Campaign has highlighted major gaps knowledge and provides a roadmap to advance the field of coma science with the goal to support the recovery of patients with DoC.

## Background

Disorders of consciousness (DoC) are frequently encountered in both, acute and chronic brain injuries [1–3]. A recent study revealed a point prevalence of coma ranging from 7 to 31 per 100,000 and an annual incidence of 135 to 258 per 100,000, based on cohorts identified using a crowdsourcing approach in the United Kingdom and the United States [4]. Overall, coma carries a poor prognosis, including death and prolonged coma [5–7] but increasingly survival and long-term recovery of function are seen. DoC does not only impact patients but also affects families, manifesting in both physical and mental health issues such as anxiety and depression [8, 9]. Major societal implications of DoC have to be considered. For instance, the direct lifetime care cost for a patient with DoC from traumatic brain injury (TBI) in the US is estimated to be around $1.5 million or more [10]. Research on DoC is essential as misdiagnosis and practices of prognostication can have profound implications for medical decision-making and health-care systems.

Accurately predicting long-term clinical outcomes and ensuring continuous, optimal care for patients with DoC are significant challenges. Difficulties arise from major knowledge gaps surrounding DoC, which is attributable to a lack of large-scale data, early withdrawal of life-sustaining treatments (WLST), inequities in access to care, uncertainties regarding recovery trajectories, inaccurate early prognostication, and variable perspectives on meaningful recovery [11, 12]. DoC exists on a temporal continuum, which alters the principles and confounders involved in evaluation and treatment over time, providing additional challenges for prognostication [13, 14].

Despite these limitations, recently, significant advancements in understanding the trajectory, prognosis, and mechanisms underlying DoC have been made, offering new hope for meaningful recovery for these patients [11]. This body of work has yielded an initial understanding of the mechanisms underlying DoC [11], outlines approaches to achieve better prognostic accuracy [1], and suggests a number of potential therapeutic approaches to be more comprehensively studied in the future [15].

In this review, we will discuss concepts of consciousness and DoC and delve into their pathophysiology. We will explore effective strategies for management and therapeutics in both acute and chronic phases of DoC and discuss the assessment of long-term outcomes within the context of acute care. Lastly, we will address research challenges and contemplate future directions of DoC investigation.

## Prognostication and withdrawal of life-sustaining treatments in disorders of consciousness

Increasingly delayed recovery of consciousness and function are seen in unconscious patients with severe brain injury, such as TBI [16–19]. However, trajectories of recovery differ from patient to patient and identifying for an individual which trajectory they are on is challenging [18]. Impairment of consciousness is an essential component of many prognostication scales, such as patients with intracerebral hemorrhage (ICH) [20], TBI [21], or cardiac arrest (CA) [22–24]. However, these scales have major limitations and are by many increasingly abandoned in clinical practice [25]. One of the major challenges to achieve more precise predictions is the confounding effect of WLST, which is commonly practiced in patients with DoC from acute, severe brain injury. Decisions about the aggressiveness of care including continued life-sustaining treatments are made based on discussions between surrogates such as family members and the treating physicians. Guiding principles are the individual patient’s expressed wishes, concepts of meaningful recovery, and predicted outcomes. The practice of WLST varies across different neurological injuries and lacks standardization, resulting in differing frequencies of WLST implementation.

In patients with severe TBI, a retrospective multicenter study reported that amongst patients that died, 70% had undergone WLST ranging from 45 to 87% across different medical centers [26]. In patients with ICH, one study demonstrated that 26% of patients underwent WLST, and of those, 51% of patients underwent WLST within two days of presentation [27]. In CA, one-third of patients who died in-hospital had WLST within 72 h of the event. When extrapolated, it is estimated that about 2300 Americans die within 72 h of the event annually in the context of WLST. By eliminating early WLST, the authors proposed that up to 64% of these patients (around 1500 patients) could potentially achieve a functionally favorable recovery (modified Rankin Score ≤ 3) [28]. There is a significant gap in our understanding of which patients may recover and what the predictors of poor outcomes are [12]. Decisions trees may help achieving a more uniform approach for WLST and support the unmasking of unconscious biases [29]. Providers further need to recognize the physical, emotional and financial implications of survival with persistent DoC and the high subsequent mortality risk of those who initially survive DoC [30, 31].

In many Japanese hospitals, WLST is rarely offered by physicians and even less frequently chosen by families as the goals of care [32]. Amongst terminally ill patients in Japan, withdrawal of mechanical ventilation occurred only in 10% of cases [33]. An international survey revealed that only one-third of Japanese physicians would issue a do-not-resuscitate order in end-of-life situations [34]. The report further suggested that almost half of the physicians in Japan continued full support for patients in a vegetative state who develop septic shock. Despite a shift towards changing this practice, WLST based solely on DoC remains uncommon in Japan, especially for patients with isolated neurological disease. One study in Japan investigated long-term outcomes of unconscious patients with ischemic or hemorrhagic stroke that within 4 days of the injury were deemed to have no chance of meaningful recovery [35]. Almost half of these patients had died within six months of the injury, but others showed unexpectedly good outcomes. One patient returned to pre-injury levels of activity, and three were able to live at home. These results gathered in a context without commonly practiced WLST further highlight the challenges of accurate prognostication.

## Consciousness

Philosophers have approached the problem of consciousness from a number of different angles but for practical purposes and the clinical context the one coined by Plum and Posner remains useful. This stipulates consciousness as a state of full awareness of the self and one's relationship to the environment [36]. Core dimensions of consciousness are “arousal” and “content” [37], which at the bedside are judged by the subject’s responses to the assessor. Limitations of this concept will be discussed below.

“Arousal” which represents the level of alertness, or wakefulness is supported by the ascending reticular activating system (ARAS) and the cortex. Specific neurotransmitters (glutamine, noradrenaline, acetylcholine, dopamine, serotonin, histamine, orexins, and gamma-aminobutyric acid) have been identified that are involved in projections from brainstem structures to the cortex in support of arousal [38].

“Content,” which represents awareness as well as cognitive and emotional reactions is regulated by distinct cortical or subcortical anatomical networks. Therefore, impairment of consciousness may be attributed to both widespread cortical impairment as well as injury to the specific brainstem and diencephalic pathways that regulate overall cortical function [36].

## Disoders of consciousness

DoC are characterized by alterations in arousal and/or content processing and may result from global pathology or focal brain injuries that induce widespread functional changes [1, 11]. Primary causes of these conditions include TBI, ICH, subarachnoid hemorrhage (SAH), acute ischemic stroke, CA and encephalitis. Clinically, DoC are primarily classified based on observable behavioral characteristics and likely exist on a continuum with frequent fluctuations between states. Patients who deteriorate or recover may progress sequentially through different states of consciousness [1]. Current guidelines specify the initial 28 days post-injury as the acute phase of DoC with the subsequent period labeled as subacute-to-chronic [16].

**Fig. 1:**
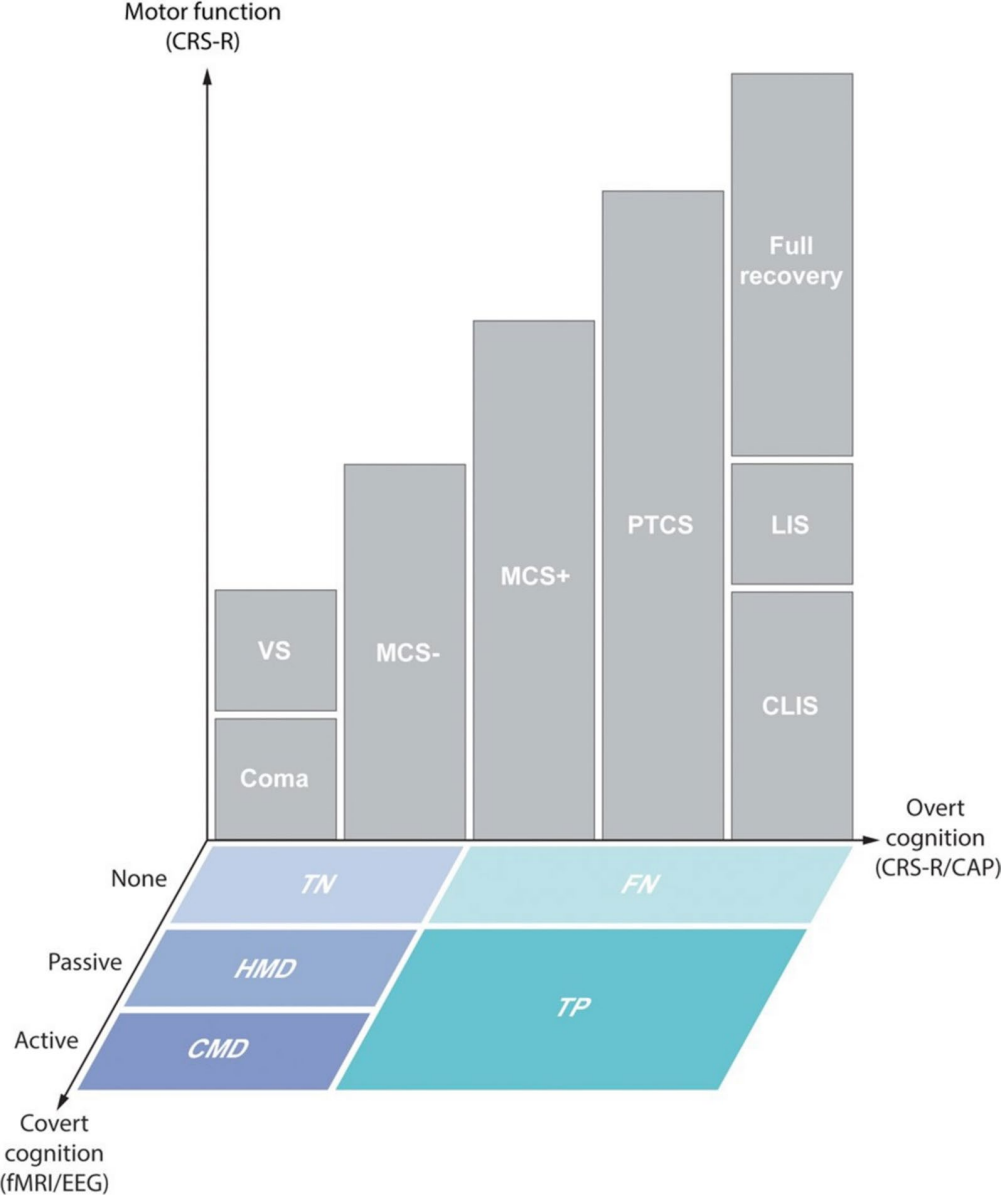
Classification of consciousness by three dimensions. Patients are categorized along three dimensions: overt cognition (*X*-axis) assessed with the CRS-R or CAP, motor function (*Y*-axis) evaluated with the CRS-R during bedside behavioral evaluations, and covert cognition (*Z*-axis) that cannot be detected by behavioral evaluations and is assessed through fMRI and EEG. Levels of consciousness, as indicated by overt cognition, range from “coma and VS/UWS,” MCS minus (absence of language function), MCS plus (presence of language function), PTCS, and “CLIS, LIS, to full recovery” of consciousness. These levels are further differentiated by motor function. In cases of coma, VS/UWS, and MCS minus, patients demonstrating active responses on fMRI or EEG can be diagnosed with CMD. If patients exhibit fMRI and EEG responses within the association cortex during passive language or music stimuli, they are considered in this approach of visualizing conscious states to have HMD status. Patients lacking behavioral evidence of language ability (coma, VS, and MCS−) are classified as TN in the absence of fMRI or EEG responses. Patients with behavioral evidence of language capability (MCS+, PTCS, CLIS, LIS, and full recovery) are classified as FN if there are no corresponding fMRI or EEG responses, and as TP if such responses are present. VS: vegetative state; UWS: unresponsive wakefulness syndrome; MCS: minimally conscious state; PTCS: post-traumatic confusional state; CLIS: complete locked-in syndrome; LIS: locked-in syndrome with preservation of minimal motor function; CRS-R: Coma Recovery Scale-Revised; CAP: Confusion Assessment Protocol; CMD: Cognitive motor dissociation; HMD: Higher-order cortex motor dissociation; TN: true negatives; FN: false negatives; TP: true positives; fMRI: functional MRI; EEG: electroencephalographic. Adapted from Edlow et al. [52]

There are several levels of DoC according to the pattern of injury impacting arousal and awareness (Fig. 1).

- ‘Coma’ is a state of sustained unconsciousness in which the eyes remain closed with a complete absence of arousal and content [39].
- ‘Unresponsive wakefulness syndrome (UWS)’ also referred to as ‘vegetative state (VS)’ is defined as arousal without behavioral evidence of awareness of self and the environment [40–42]. Chronic VS refers to patients who persist in this state for more than 12 months following TBI, or for more than 3 months following non-TBI [16]. It is important to note that behavior observed in VS, like sleep–wake cycles, blinking, and the startle reflex, are not indicative of conscious intent and don't reflect an awareness of oneself, others, or the environment [40].
- ‘Minimally conscious state (MCS)’ is a condition of severely altered consciousness in which there is minimal and inconsistent but definite reproducible behavioral evidence of awareness of self or environment [43]. There are two dimensions for MCS. In MCS+, language-related behaviors, such as language comprehension and/or expression, are preserved [44]. On the other hand, patients with MCS- may track or attend to the examiner, but don’t show any evidence of functioning comprehension.
- States of consciousness between MCS and full recovery. The various states of higher functioning DoC have been given a number of labels and classifications [45], including emergence from MCS [43], post-traumatic amnesia [46], traumatic delirium [47], and posttraumatic confusional state (PTCS) [48]. The 'confusional state (CS)’ has been defined specifically for TBI based on the criteria from the Confusion Assessment Protocol [11, 49]. The CS should be considered within the spectrum of DoC as it is characterized by persistent global cognitive disfunctions, behavioral dysregulation, attentional abnormalities, disorientation, disrupted sleep/wake cycle, fluctuating symptoms, and consequently altered consciousness [11, 48]. Locked-in syndrome must be clearly distinguished from these states, as it is characterized by anarthria and quadriplegia with out-of-proportion preserved cognition [27, 36].
- Covert consciousness: Traditional assessments of consciousness have focused on overt cognition or in other words relied on assessing consciousness based on the patient’s motor response to verbal motor commands (Fig. 1). Over the past two decades it has become clear that a dissociation between relatively intact cognition and lack of external expression can be detected [50]. This state is characterized by intentional brain activity that can be detected by Magnetic Resonance Imaging (fMRI) detecting blood flow or electroencephalography (EEG) detecting neuronal activity, both recorded following the presentation of a motor command [51]. This state can be detected in patients whose clinical presentation may align with conditions like coma, VS/UWS, or a minimally conscious state MCS− [52]. A number of labels have been assigned to this state and related phenomena, but most commonly the term covert consciousness or cognitive motor dissociation (CMD) is used and we will use CMD in this review.

## Mechanism of consciousness and pathophsyiology of DoC

Stupor or coma results from extensive injury to both cerebral hemispheres or more “strategically placed” smaller lesions, for example in the ARAS [36]. The ARAS is integral to initiating and sustaining a state of wakefulness. Coma can be characterized as a disruption in this extensive neural network, which encompasses significant areas of the dorsal upper pons, midbrain, and thalamus, extending to the cortex of both cerebral hemispheres [36]. Neuronal clusters located within the tegmentum of the pons and midbrain, the intralaminar nuclei of the thalamus, and the posterior hypothalamus have established connections with the basal forebrain and its related cortical areas [53]. Previously thought to be a singular entity, the activation system is currently understood to consist of multiple, interconnected networks. These regulate cortical activity by employing projections that utilize neurotransmitters such as acetylcholine, noradrenaline, serotonin, and dopamine. The influence of these projections reaches the cerebral cortex not only directly but also through the thalamus and various other pathways [54]. In contrast, unilateral hemispheric lesions or those located at the level of the mid-pons or below in the brainstem generally do not typically result in coma. The predominant pathophysiological principal underlying this dysfunction involves a broad decline in excitatory synaptic activity.

Injury location provides a crude initial approach to classify a large number of potential underlying pathologies into three main categories (nonstructural psychiatric causes of DoC should be recognized but are disregarded here for the purposes of classifying lesions based on injury location).

- *Supratentorial structural lesions* involving bilateral cerebral hemispheres and/or diencephalic regions. Classic conditions associated with this include bilateral large hemispheric strokes and severe traumatic brain injury.
- *Infratentorial structural lesions* impacting the ARAS in the brainstem may be seen in conditions such as hemorrhages in the brainstem or extensive cerebellar hemorrhages causing mass effect on the brainstem.
- *Non-structural or metabolic pathologies* leading to DoC may be seen in conditions such as hypoglycemia, hyponatremia, and cardiac arrest or hypoxia. In unselected cohorts of patients with DoC admitted to an emergency room these patients represent the majority of cases.

### Theories of consciousness

Neural correlates of consciousness continue to be debated and no agreement has been reached as to which theory best explains consciousness. Two frequently debated ones include the global neuronal workspace (GNW) and integrated information theory (IIT). GNW postulates that neuronal activity results in activation of interconnected higher-order areas resulting in global broadcasting of information [55]. Critical components of this concept include local, specialized cortical processors, which are centrally linked via interconnected areas over long distances and specialized modular processors, which work unconsciously (e.g., “long-term memory,” “evaluative systems,” “attentional systems,” and “perceptual systems”). The late evoked potential, also known as P300b [56], is associated with a conscious response to a sensory stimulus and can be conceptualized within this context [57]. IIT on the other hand states that the complexity of the physical substrate of consciousness is contingent on causal interactions between elements of the system [58] and a high degree of integrated information or consciousness are reached due to abundant causal relations of subsets of the system. The level of integrated information as a correlate for quantifying consciousness can be measured by recording and analyzing the EEG signal for example to a transcranial magnetic stimulation (TMS) stimulus in a predefined cortical area, as realized in the calculation of the perturbational complexity index (PCI) [59]. Both, P300b and PCI as measures of consciousness are at this point primarily tested outside of the acute brain injury context but may have practical applications for ICU care as a more objective measure of consciousness in the future.

### Models and theories underlying disorders of consciousness

#### Mesocircuit model (Fig. 2)

**Fig. 2:**
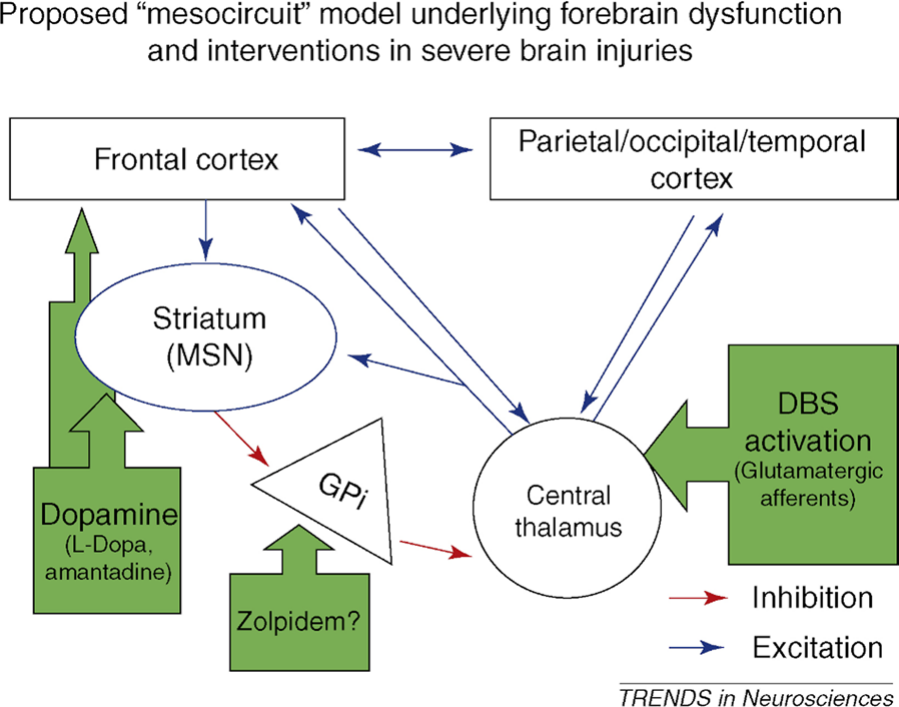
The mesocircuit model. This figure illustrates a mechanism for downregulation of the anterior forebrain mesocircuit in severe acute brain injuries [60]. Following acute brain injury, reduced output from the thalamus to the cortex and striatum leads to a decrease in inhibitory input to the GPi. The diminished inhibitory output from the striatum permits neurons in the GPi to continuously activate, exerting sustained inhibition on other neurons, including those in the already heavily suppressed central thalamus. This mesocircuit model is useful for conceptualizing potential mechanisms of treatments aimed at restoring consciousness in cases of severe acute brain injuries. Both tDCS and rTMS can activate the frontal cortex and modulate cortical excitability [208, 209]. Additionally, rTMS has the potential to globally increase cortical oscillations when applied over the primary motor cortex. Dopamine (l-Dopa, amantadine) may activate both the frontal cortex and the striatum, enhancing fronto-parietal brain metabolism [62]. Zolpidem is thought to inhibit the GPi, which may subsequently activate the frontal cortex and striatum [63, 64]. DBS acts directly on the central thalamus, targeting the enhancement of thalamo-cortical connectivity [65]. LIFU also non-invasively stimulates the thalamus [199]. VNS, whether invasive or non-invasive, directly stimulates the vagal nerve, influencing brainstem activity [210, 211]. The ellipses, triangle, and circle represent subcortical regions, and the rectangles denote cortical areas. MSN: medium spiny neurons; GPi: globus pallidus interna; tDCS: transcranial direct current stimulation; rTMS: repetitive transcranial magnetic stimulation; DBS: deep brain stimulation; LIFU: low intensity focused ultrasound pulse; VNS: vagus nerve stimulation. Adapted from Schiff [60]

Figure 2 introduces a proposed 'mesocircuit' model illustrating the susceptibility of the anterior forebrain to multifocal brain injuries, leading to extensive deafferentation or neuronal loss [15]. This model emphasizes the critical role of thalamocortical projections for patients with DoC [60], with major theoretical implications providing a framework to conceptualize treatment approaches. Thalamocortical projections are identified as significant activators, strongly influencing both cortical and striatal neurons. The model highlights the dependency of medium spiny neurons (MSN) in the striatum on high background synaptic activity and dopaminergic neuromodulation to sustain firing rates. Without MSN output, the globus pallidus interna may inhibit the central thalamus, potentially initiating an anterior forebrain shutdown. The model also provides a theoretical framework to explain a number of interventions such as dopaminergic agents [16, 61, 62], zolpidem [63, 64], and electrical brain stimulation of central thalamic nuclei [65, 66].

## Predicting recovery of consciousness

**Fig. 3:**
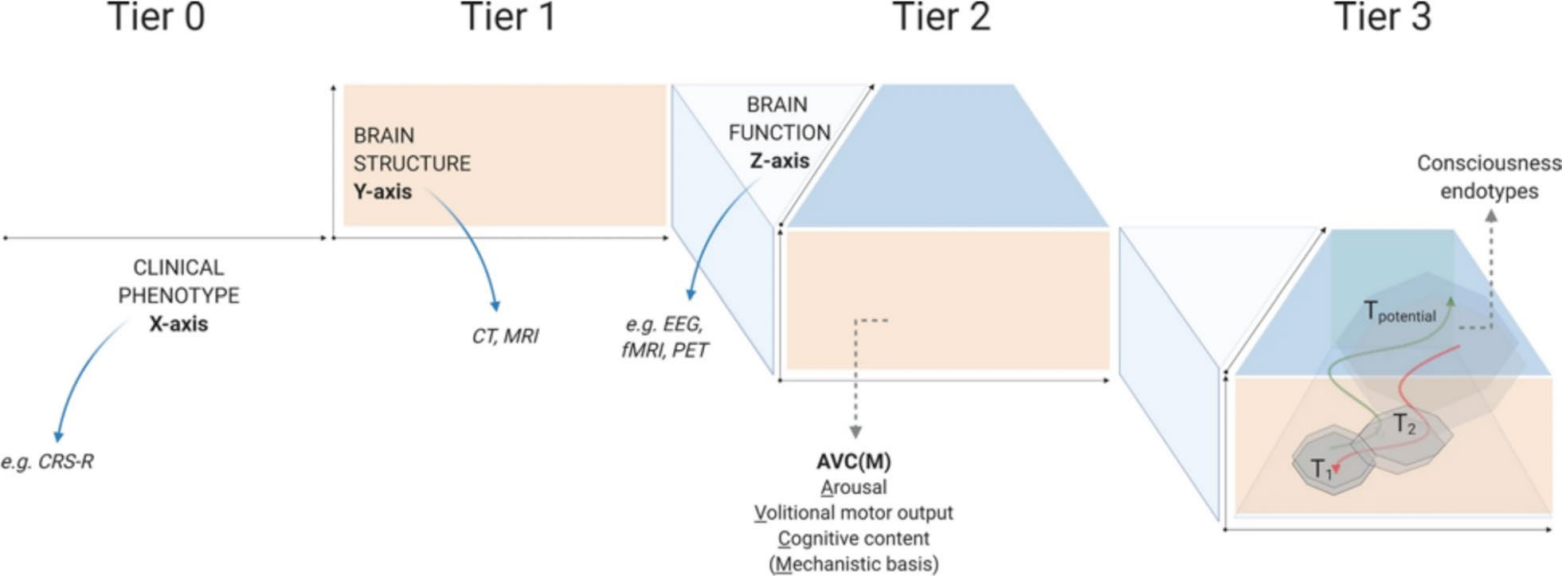
Advanced Classification of Consciousness Endotypes (ACCESS). This figure delineates a novel, three-tiered model (Tier 1, Tier 2, Tier 3) for assessing consciousness. Tier 1, suitable for resource-limited settings, focuses on clinical evaluations (e.g., CRS-R) and structural neuroimaging techniques (CT, MRI). Tier 2 extends to include functional MRI and EEG to identify covert consciousness in non-responsive patients (CMD). Tier 3 integrates extensive biological and physiological data, facilitating longitudinal monitoring of clinical trajectories for consciousness and the evaluation of novel therapeutic interventions. CRS-R: Coma Recovery Scale–Revised; CT: computed tomography; EEG: electroencephalography; fMRI: functional magnetic resonance imaging; MRI: magnetic resonance imaging; CMD: cognitive motor dissociation; PET: positron emission tomography;T1 and T2: time points 1 and 2. Adapted from Kondziella et al. [75]

Our ability to predict recovery of consciousness, recovery of crude neurological function, and patient-centered outcomes is poor [67–72]. To improve our ability to predict recovery, one approach is to utilize the concept of pheno- and endotypes as sub-groups of patients have been demonstrated to have a distinct shared trajectory [12, 69, 73, 74]. This involves gathering detailed information to better characterize the patient and applying population data in a way that incorporates these personalized characteristics to make an individualized prognosis (Fig. 3, Advanced Classification of Consciousness Endotypes (ACCESS) [75]. This approach, akin to personalized medicine, may allow us to better forecast a patient's recovery trajectory. The framework is designed to adaptively integrate advancements in molecular and cellular research with the goal of more comprehensively capture underlying mechanisms of brain function, referred to as consciousness endotypes [75].

### Physical examination for predicting the recovery of consciousness ('tier zero')

The foundational level, tier zero in the ACCESS framework, for predicting recovery of consciousness, relies on bedside behavioral assessments. Common tasks might include instructing the patient to follow specific commands according to standardized assessments. Historically, the Glasgow Coma Scale (GCS) was introduced as an initial measure, primarily for TBI patients [39]. The GCS is widely used and categorizes eye-opening, verbal and motor responses. However, its ability to assesses consciousness remains limited. The Coma Recovery Scale-Revised (CRS-R) is the gold standard for the assessment of consciousness [76]. While the CRS-R has certain limitations, including ceiling effects and limited use in the ICU due to its lengthy administration time, it offers a robust and standardized approach to assessing consciousness [77–79]. The abbreviated version of the CRS-R, the CRS-R For Accelerated Standardized Testing (CRSR-FAST) was developed, enabling administration time was approximately one-third of the full-length CRS-R. It has demonstrated a sensitivity, specificity, and accuracy for detecting consciousness of 81%, 89%, and 84%, respectively in an initial study but to date has not been widely tested. The CRSR-FAST facilitates repeated assessments of consciousness, which are essential for accurate diagnosis and prognostication [79].

### Structural assessments for predicting recovery of consciousness ('tier one')

In tier 1 of the ACCESS framework, structural assessments including techniques such as Computerized Tomography (CT) imaging and MRI are widely used to better classify the current state of patients and their potential for recovery of consciousness and outcomes. Structural imaging may identify underlying causes for impaired consciousness guiding management decisions (i.e., herniation triggering a neurosurgical procedure in a patient with traumatic brain injury) [80] or providing information to inform prognostic assessments (i.e., the volume or location of an intracerebral hemorrhage).

A comprehensive overview of the many ways that various forms of imaging can inform assessments and predictions in patients with DoC is beyond the scope of this review. A number of widely available MRI sequences, including diffusion-weighted imaging [81–83], susceptibility-weighted imaging, and T2*-weighted imagine [84–87], are used to further assess underlying pathophysiology in clinical practice [11, 88, 89]. However, the role of these to support prognostications is less certain and confounders need to be considered [86, 90–92].

Diffusion tensor imaging (DTI) allows visualization of the microstructural integrity of white matter tracks and provides a means to explore the structural connectivity between brain regions [93]. One study demonstrated that in patients who remain unconscious 7 days following a CA, the normalized quantitative whole-brain white matter fractional anisotropy value, as measured by diffusion tensor imaging, could serve as a precise predictor of neurological outcomes at 6 months [94]. Similarly, in TBI patients global measures of microstructural white matter injury derived from DTI show promise for improving predictions [95–98]. Technically more challenging is the reconstruction of axonal fiber tracts based on DTI sequences in patients with large structural lesions [99–101]. This approach does have the potential to unmask underlying mechanisms of impaired consciousness and potentially even direct intervention to support recovery. The approach has been successfully applied to visualize impaired white matter tracts of the ascending arousal network in patients with DoC [92, 102], providing first insights into treatment approaches for these patients [103]. Please refer to the following for a more comprehensive discussion of neuroimaging as it applies to patients with DoC [104–107].

### Assessments of brain function ('tier two')

In tier 2 of the ACCESS framework, measures of brain function are utilized, which are recorded from the brain at rest or in response to external stimuli and interventions.

### Resting state EEG

Resting EEG (rEEG) evaluates the brain while the patient is at rest or in other words not exposed to a repeated, standardized stimuli. In the critical care setting primarily, rEEG is used to detect seizures, a potentially reversible cause of DoC. It can further be used as a monitor for brain ischemia [108–111], another potential cause for impaired consciousness.

More directly, EEG patterns have been correlated with different states of consciousness and correlations have been made between EEG patterns and recovery of consciousness. This prospect has been greatly supported by computational analysis of the digitally recorded EEG, also known as quantitative EEG [112]. Computational analysis of the EEG signal allows rapid assessment and provides summary statistics of long EEG recordings which is crucial for the study of DoC as consciousness is not static and may rather fluctuate [113]. For these purposes, raw EEG waves can be decomposed into distinct waveforms, by for example using Fast Fourier Transform. A large number of different qEEG metrics can be generated that explore different statistical properties of the EEG signal. In the following, we will focus on some of the more frequently investigated EEG metrics.

### Commonly used qEEG metrics

**Fig. 4:**
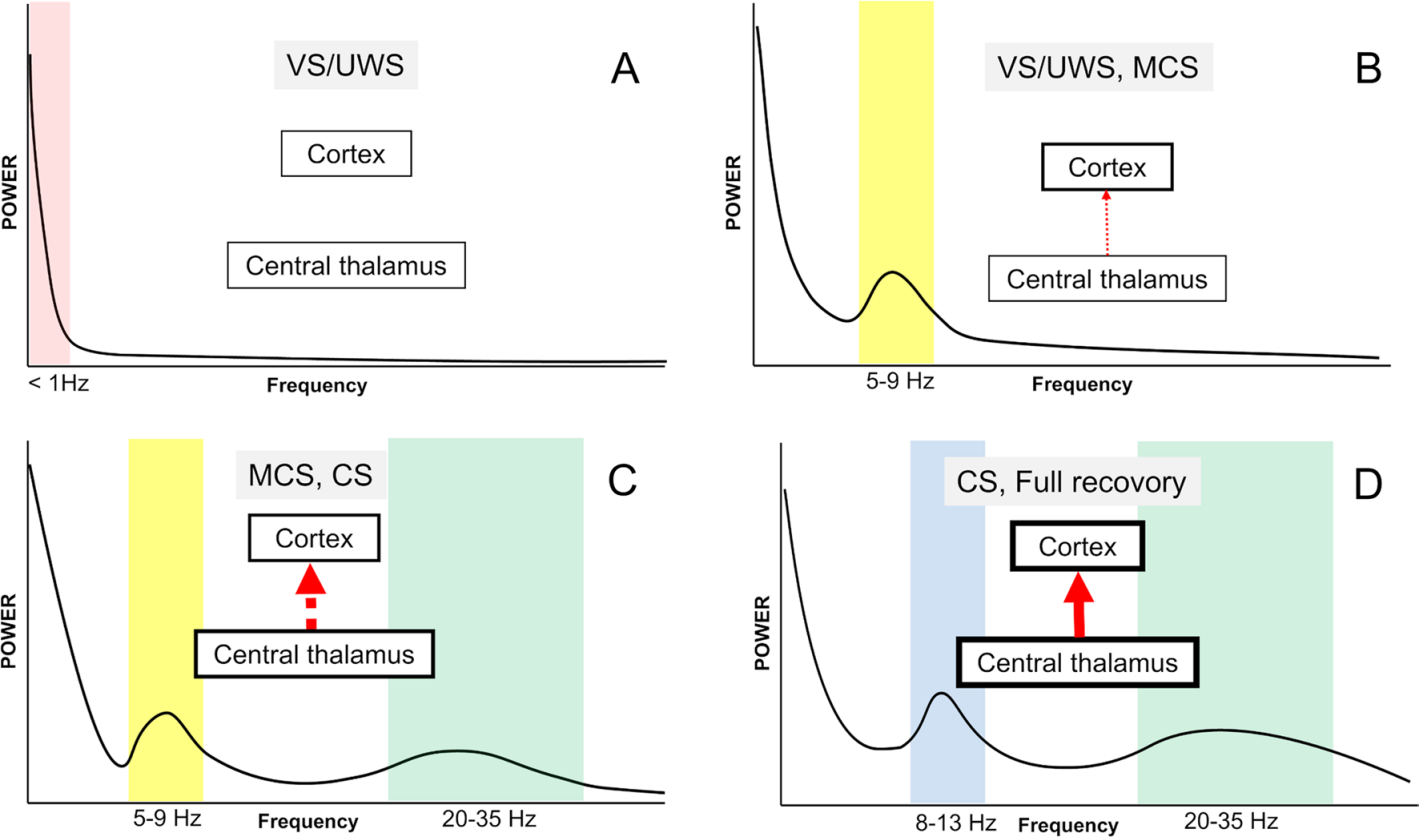
ABCD categories with their respective characteristics for thalamocortical status and clinical signs. The 'A' category, with a dominant frequency of less than 1 Hz, indicates a thalamocortical network that is completely disconnected. Neocortical neurons are significantly hyperpolarized and primarily generate low-frequency oscillations [212]. The likely behavioral diagnosis is VS/UWS [116]. In the 'B' category, where the dominant frequency is ~ 5–9 Hz, the thalamocortical network is severely disconnected (indicated by a thin red dotted line). The intrinsic oscillation of membrane properties in neocortical neurons results in bursts at a rate of about 5–9 Hz due to depressed membrane potentials[213]. The behavioral diagnosis could be VS/UWS or MCS [11]. The 'C' category, characterized by dominant frequencies of approximately 5–9 Hz and 20–35 Hz, corresponds to a moderately disconnected thalamocortical network (indicated by a red dotted line). Partial restoration of neocortical membrane potentials, along with the coincident bursting of deafferented thalamic neurons, leads to the coexistence of theta and beta frequency oscillations in the connected cortex. The behavioral diagnosis would most likely be MCS or CS[11]. The category 'D', with dominant frequencies of approximately 8–13 Hz and 20–35 Hz, signifies a fully intact thalamocortical network. Normal firing patterns of neocortical neurons, coupled with a structurally and functionally interconnected thalamus and cortex (indicated by a red arrow), facilitate the production of alpha and beta frequency oscillations. The behavioral diagnosis could be CS or healthy. VS/UWS: vegetative state/unresponsive wakefulness syndrome; MCS: minimally conscious state; CS: confusional state

**Fig. 5:**
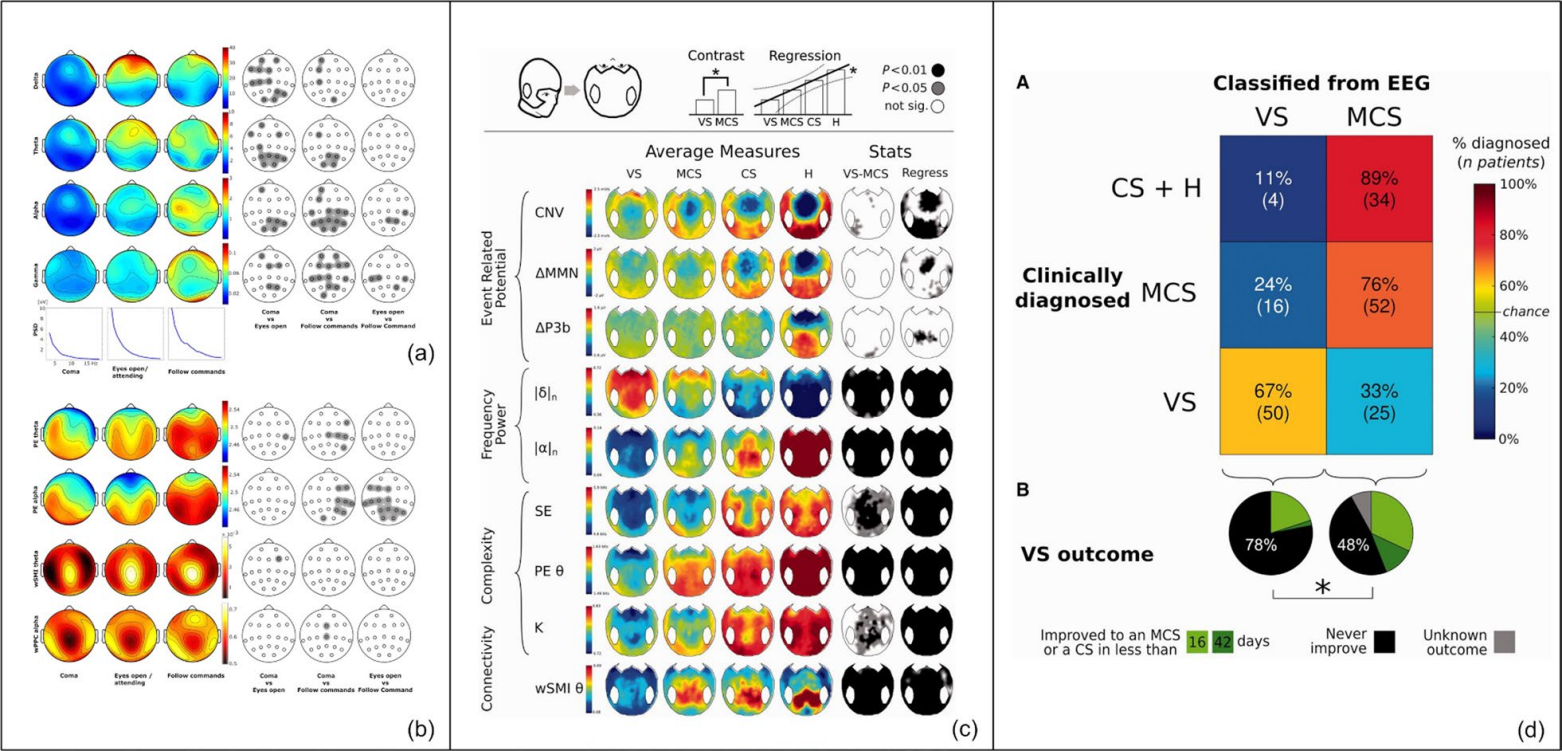
Quantitative EEG analysis of resting state EEG in patients with acute and chronic disorders of consciousness. **a** Stratification was based on three behavioral assessments: coma, eyes open/attending, and following commands. Spectral power plots (rows 1–4) and Posterior PSD plots (row 5) were analyzed accordingly. Statistical analyses were carried out to assess the grouped effects at each electrode among different behavioral states, with gray in the right three columns indicating *P* < 0.05. An increase in diffuse delta (row 1) and posterior theta (row 2) was observed between coma and arousal states. Progressively increasing posterior alpha (row 3) and central gamma (row 4) were evident between assessments consistent with coma, arousal, and awareness. Higher levels of consciousness were associated with an overall increase in power across all frequency bands, as indicated by the posterior PSD plots (row 5). **b** Complexity and coherence measures included PE theta (row 1), PE alpha (row 2), wSMI theta (row 3), and wPPC alpha (row 4), which correlated with the three best behavioral assessments stratified by time. wSMI theta and wPPC alpha, as connectivity measures, are represented using distinct color maps. Statistical analyses to compare the grouped effects at each electrode across various behavioral states are presented in the right three columns, where grey indicates *P* < 0.05. In complexity measures, theta and alpha frequency PE were significantly higher in patients who were aware, especially in the parieto-occipital regions for alpha frequencies (rows 1 and 2). Regarding information-sharing measures, slight differences in wSMI theta (row 3) were noted among the behavioral states. WPPC in alpha frequencies increased from coma to awareness (row 4), with a significant rise in central channels in aware patients compared to those in coma. **c** A comparison of SAH data (**a**, **b**) with data from patients with chronic disorders of consciousness due to TBI reveals that the frequency power of the alpha (|α|_n_) and theta PE in VS and MCS is similarly distributed to those in the acute phase of SAH. **d** On the *x*-axis, the prediction based on EEG was shown as VS or MCS. On the *y*-axis, the clinical diagnosis was shown as VS, MCS or CS/Healthy. Each cell provides the count of recordings along with their corresponding percentages for each clinical state category. In most instances, EEG-based classification aligns with the clinical diagnosis for patients with VS and MCS. The pie charts illustrate the clinical outcomes for patients clinically diagnosed with VS, based on whether EEG assessments categorized them as VS or in a higher state of consciousness such as MCS or CS. As shown in green, the probability of improvement was significantly higher in MCS patients diagnosed with EEG (*P* = 0.02). PSD: power spectral density; PE: permutation entropy; wSMI: weighted symbolic mutual information; wPPC: weighted pairwise phase consistency; SAH: subarachnoid hemorrhage; TBI: traumatic brain injury; CNV: contingent negative variation; MMN: mismatch negativity; ΔP3b: P300b; |δ|_n_: normalized power in delta band; |α|_n_: normalized power in alpha band; SE: spectral entropy; PEθ: permutation entropy in theta band; K: Komolgorov-Chaitin Complexity. EEG: electroencephalogram; VS: vegetative state; CS: conscious state. Adapted from Claassen et al. [153] and Sitt et al. [122]

- Power: This relates to the amplitude of the EEG signal and can be analyzed as a total (i.e., across all frequencies) or for specific frequency bands (i.e., limited to the theta frequency range between 4 and 8 Hz). Power can be visualized over time (i.e., using density spectral arrays) or as spatial maps to represent the distribution on the surface of the brain [114]. Power spectral density plots represent the power across difference frequencies averaged over a selected time range. The ABCD model (Fig. 4) classifies EEG spectrograms on a scale from A to D based on the presence of visible power peaks in specific frequency ranges using power spectral density plots [11]. ABCD categories relate to patients with impaired consciousness and reflect the extent of underlying thalamocortical deafferentation [115]. Pattern A correlates with severe disconnection between the thalamus and cortex and EEG power is severely diminished across all frequency ranges. Pattern B corresponds with moderate thalamocortical disconnection with re-emergence of the theta frequency peak. As connectivity increases, a beta peak may emerge as seen in Pattern C. With mostly intact connectivity between the thalamus and the cortex with an alpha peak and a beta peak (Pattern D) [11].
- Outcomes in comatose patients following cardiac arrest [116, 117] and subarachnoid hemorrhage [118] correlate with the ABCD framework. During recovery from DoC, patients may transition through different ABCD categories [117], initially exhibiting lower-level patterns, akin to those categorized as Type A or Type B, and then transitioning to patterns C or D, as recovery progresses. This suggests that thalamocortical disconnection is not necessarily structural but can also be functional.

- Functional connectivity (Fig. 5): Assessments of EEG synchronicity in the frequency and amplitude domains between different recording sites on the brain can provide measures of functional connectivity. A number of these such as coherence [119, 120] or weighted symbolic mutual information (wSMI) [121, 122] have been used widely in neuroscience and in patients with DoC [123]. wSMI evaluates the extent to which two EEG signals exhibit nonrandom joint fluctuations, suggesting intact functional connectivity supporting information sharing between two regions [124].
- Complexity (Fig. 5): Complexity of the EEG signal can be analyzed in a number of ways, including permutation entropy (PE) [125–128]. In short, PE first transforms a time series into a sequence of discrete symbols of a given length before estimating the entropy of the resulting distribution of sequences. Derived from EEG it can serve as a measure of local information content [129–131] with relevance to information processing within local and widespread cortical networks [128]. In patients with DoC PE in specific frequency bounds is altered and may provide additional qEEG metrics to track DoC [122].

### Resting EEG in patients with DoC

The potential of qEEG analysis for tracking behavioral states and predicting recovery for patients with DoC is supported by a large body of evidence, including studies in patients with acute and chronic DoC [64, 116, 122, 132] and anesthesia-induced reversible loss of consciousness [133–136]. It is still an ongoing debate about what rEEG frequency (i.e., slow or faster frequencies) or feature (dominant frequency vs complexity vs functional connectivity measures) most closely maps with consciousness [137].

Structural injury to the brain such as that seen in patients with brain tumors or strokes results in focal slowing on EEG and diffuse background slowing is seen in patients who receive sedatives and those who are encephalopathic [108, 138–142]. In line with these observations, high amplitude delta oscillations have traditionally been considered as a signature of impaired consciousness, but recently a number of studies called this into question. Prominent delta frequency oscillations have in contrast to the traditional interpretation been associated with conscious states, including in studies with propofol-induced loss of consciousness and delirium [143–145]. On the other hand, in healthy conscious patients, alpha power and coherence in the alpha frequency range are prominent in posterior brain regions [146].

Higher EEG complexity has been reported in MCS patients with chronic DoC when compared to patients with chronic VS or acute coma [147]. In patients undergoing propofol anesthesia, power and coherence in the alpha frequency range shifts from the occipital to frontal brain areas as patients lose consciousness and relocates to the back of the brain during emergence from anesthesia [136, 146, 148–152].

Theta frequency range PE has been used to distinguish patients with intact from impaired consciousness early after SAH [153] and sub-acutely after TBI [122]. A similar finding can be observed in cardiac patients with recovery of command-following [130].

Important considerations that affect the EEG signal are medications, artifact, and biological variables such as age and sex. Elderly patients exhibited reduced alpha-band EEG power and coherence, indicative of age-dependent changes in thalamocortical function, and are more likely to experience episodes of burst suppression [154]. Depending on an infant's age, anesthetic agents can induce variations in EEG patterns, which may include changes in frequency oscillations and the presence of frontal alpha predominance and coherence [155]. Sedation and anesthetics need to be considered when analyzing EEG patterns but importantly these differ depending on the sedative or anesthetic used. Propofol is associated with highly coordinated frontal thalamocortical alpha oscillations [136] and asynchronous slow oscillations [156], and dexmedetomidine generates slow oscillations and spindle-like activity [157]. Prediction models accounting for these demographic and medication exposure factors as confounders will likely yield higher accuracy.

qEEG signatures may not only track the state of consciousness but also may bear information to predict future recovery, which would make them particularly interesting for clinical applications in the ICU. A comprehensive set of EEG markers including those derived from power metrics, complexity, and functional connectivity have been developed that associate with DoC and the potential for recovery [158]. These prediction models developed using machine learning methodologies were generalizable to an independently collected dataset from a different site. Most interestingly, these investigators also showed that patients that were “falsely” classified as being “conscious” at a time when behaviorally the patients are “unconscious” had a higher chance of recovery in the next couple of months when compared to those who were classified as being “unconscious”. In other words, in this cohort of patients mostly with traumatic brain injury that were behaviorally unconscious the EEG may have detected a signal that predicted future recovery that was impossible to detect on behavioral examination alone [122].

- Resting state MRI
  Resting-state MRI allows the examination of functional connectivity between different brain regions[159], potentially aiding in the prediction of outcomes [160]. This method scrutinizes the correlation of high-frequency fluctuations in the Blood Oxygen Level-Dependent (BOLD) signal to propose functionally connected brain regions [161]. Recovery of consciousness necessitates restoration of dynamic interactions among various subcortical and cortical networks [11]. Resting state MRI studies have implicated the default mode network (DMN), Salience Network (SN), and Executive Control Network(ECN) [162–164].

The DMN may play a role in various facets of internal or self-directed thinking, featuring central nodes located in the posterior cingulate cortex and the medial prefrontal cortex [165]. While clearly playing a role in the recovery of consciousness [163, 166, 167], recovery of the DMN alone is not sufficient for the recovery of consciousness [168]. The SN is instrumental in the detection and integration of information, possessing central nodes in the dorsal anterior cingulate cortex, and the anterior insular, and has robust connectivity to subcortical and limbic structures [169]. The ECN, containing widespread nodes in the dorsolateral fronto-parietal cortices, displays strong activation during cognitive tasks [170]. Additional contributions to recovery are believed to emerge from SN and ECN [164].

One study investigated EEG functional connectivity within these networks by analyzing pairwise coherence among EEG contacts corresponding to the networks identified in the resting-state fMRI analysis [171]. Within the right executive network, a notable increase in connectivity was observed between the Fp2 and F4 electrodes in the theta frequency range. This increase was specifically identified in patients who responded to commands, in contrast to those who did not. The 'CONNECT-ME' study, developed the multimodal approach much further to a potential prognostic tool. It demonstrated that integrating resting-state fMRI analyses with EEG resting-state approaches enhanced the accuracy to predict patient outcomes [172]. By employing machine-learning algorithms, the combined EEG and fMRI data proved instrumental in predicting consciousness levels, hinting at promising directions for future research.

## Cognitive motor disocciation

Traditionally, level of consciousness has been conceptualized as overt consciousness with motor function providing evidence of being conscious (Fig. 1). Recent studies revealed that even patients without any ability to express their consciousness with a motor act can be conscious [51]. The most widely used label for this state is CMD which can be revealed in a behaviorally unresponsive patient by means of task-based fMRI or [52] (see definition above).

### Manifestation in an individual patient

**Fig. 6:**
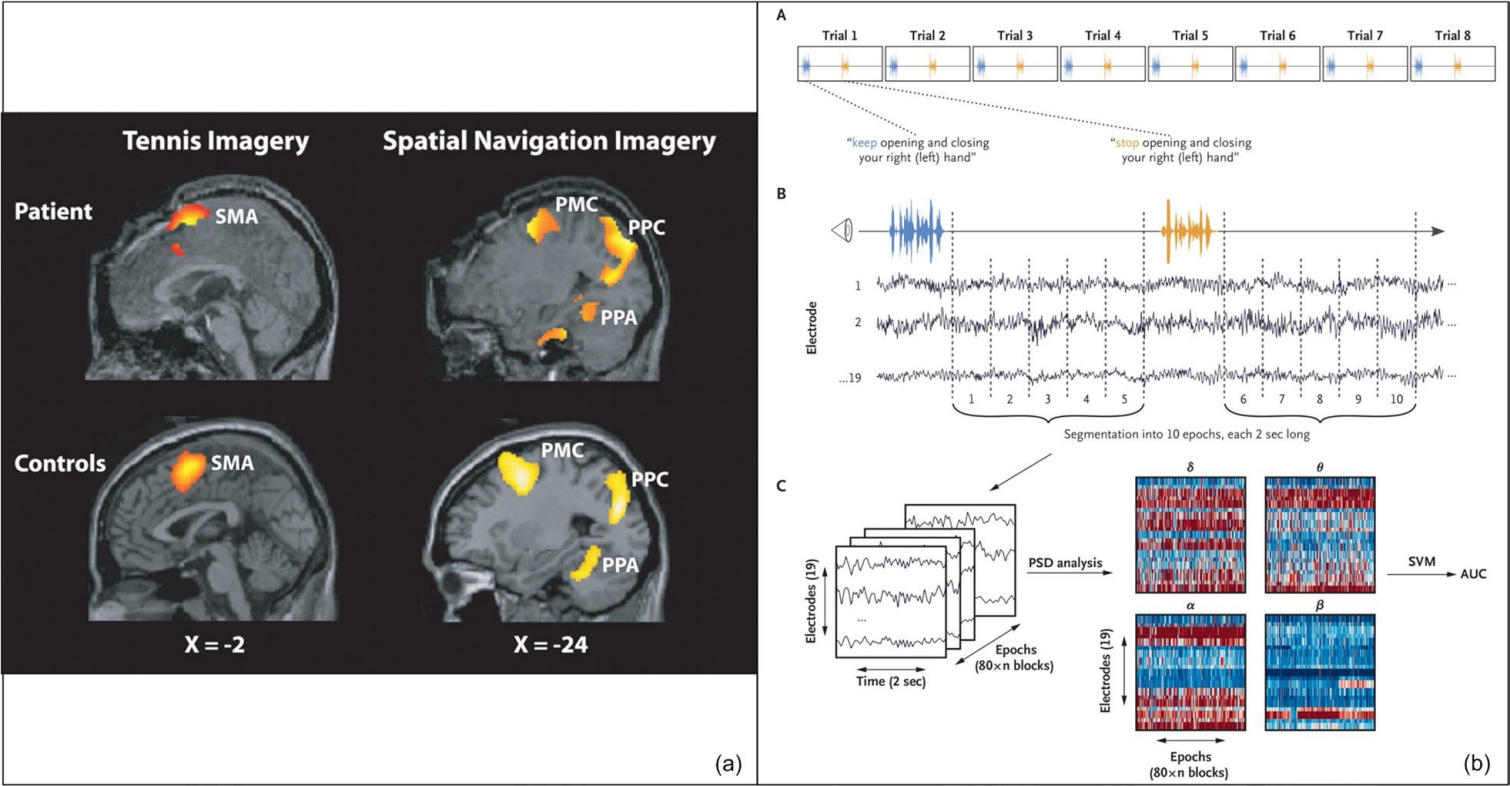
Detection of cognitive motor dissociation Using MRI and EEG. **a** fMRI of a 23-year-old woman with severe traumatic brain injury who remained in a vegetative state 5 months after the injury demonstrated similar brain activation to motor imagery tasks (top two rows) when compared to a group of healthy volunteers (bottom two rows). This brain activation was considered evidence of preserved conscious awareness in a behaviorally unresponsive patient. **b** Experimental design for EEG acquisition to detect cognitive motor dissociation in response to motor commands. EEG is recorded while patients are exposed to the motor commands “keep opening and closing your right (left) hand” alternating with “stop opening and closing your right (left) hand”. 10 s of EEG data is extracted (**A**) and segmented into 2 s epochs (**B**). Power spectral density analysis is applied in four frequency ranges: δ, θ, α, and β (**C**). Features derived from this analysis are then employed in both the training and evaluation of the support vector machine. **a** Adapted from Owen et al. [50]. **b** Adapted from Claassen et al. [175]. SMA: supplementary motor area; PMC: premotor cortex; PPC: posterior parietal-lobe; PPA: parahippocampal gyrus; PSD: power spectral density; SVM: support vector machine; AUC: area under the receiver-operating-characteristic curve

In 2006, Owen et al. investigated a young woman with a TBI who had been in a vegetative state for an extended period, placed her in an fMRI scanner and instructed her to envision playing tennis and then to think about walking through her apartment (Fig. 6a) [50]. The fMRI scanner revealed that brain regions showed distinct increases in blood flow to each task that were similar to healthy volunteers. In other words there was imaging evidence for brain activation to imagined motor tasks that in the patient despite an inability to demonstrate motor behavior [51]. This observation, known as CMD was later also revealed using computational analysis of the EEG recorded during the presentation of motor commands [173, 174]. Later, investigators demonstrated that this was not only detectable in the chronic DoC state but also early after brain injury in the ICU [52].

### The prevalence of CMD in the ICU and prognostic implications

CMD has been reported in 15–25% of unresponsive patients in the acute and subacute/chronic DoC state [175, 176]. CMD has been detected in severe acquired brain injury, such as TBI, SAH, ICH, and CA [51, 52, 175, 177] and has been found to be independently associated with a higher chance of early recovery of behavioral evidence of consciousness and good neurological function at 1 year after injury [52, 175, 177, 178].

CMD can be detected using standard 21-electrode montage EEG making it feasible to collect repeated assessments in the ICU context(Fig. 6b) [174]. EEG is recorded while patients are repeatedly presented with motor commands. For example, the motor commands “keep opening and closing your right (left) hand” and “stop opening and closing your right (left) hand” are presented via headphones to the patient while the EEG is recorded [175]. PSD analysis applied to the recorded EEG generates a dataset that is used to train a machine-learning algorithm (i.e., a support vector machine [SVM] with a linear kernel) to distinguish between the EEG responses that follow each command [175]. CMD is detected if the SVM detects a systematic difference between the two commands.

In the ICU context, CMD has been observed in 14–25% of unresponsive patients with various brain injuries and was on average detected within 4 days (interquartile range, 2–5) post-injury [52, 175, 177]. 50% of CMD patients began following clinical commands before hospital discharge compared to only 26% of those without CMD. At 12 months, 7 of 16 (44%) with CMD and 12 of 84 patients (14%) without CMD had a GOS-E level of 4 or higher [175].

Underlying mechanisms of CMD are uncertain [179], but thalamocortical projections have been implicated by a single case using DTI [180]. No single unifying structural injury underlies CMD but a structural imaging studies combined with an assessment of functional connectivity using rEEG implicated a failure of integrating comprehended motor commands with motor output with CMD. Injuries associated with impaired arousal (i.e., those affecting the ascending arousal system) and those affecting language comprehension (i.e., left thalamus) were associated with unresponsiveness without CMD [181]. These findings fit well into the anterior forebrain mesocircuit model discussed above.

Trajectories of recovery of unresponsive patients with CMD early after brain injury are different from those without CMD. One study demonstrated that CMD was an independent predictor of shorter time to good recovery [177]. Among patients discharged to home or to a rehabilitation setting, those with CMD consistently had better recovery even though they were behaviorally indistinguishable from those without CMD when the CMD detection was made.

## Management of DoC

Management of severe brain injuries in patients with DoC has historically been primarily supportive, but new data demonstrates the promise of drugs and brain stimulation even in the chronic DoC state to provide benefit years after injury [15]. Insights into specific circuit malfunction may provide opportunities for targeted interventions [11]. For example, central thalamic stimulation may be highly effective for patients with thalamic lesions [65, 182] but not universally, while amantadine administration could aid consciousness recovery in cases of injury to dopaminergic pathways [61, 183, 184].

Current experimental therapies for patients with DoC include pharmacologic, electromagnetic, mechanical, sensory and regenerative interventions (Fig. 2) [185]. While these innovations show promise, significantly more research is needed to assess effectiveness in specific patient populations [15]. The subsequent section will provide a very brief overview of commonly utilized treatment modalities for DoC.

Pharmacologic therapies for patients with DoC (Fig. 2):

Amantadine acts via several mechanisms including facilitating the output of MSN in the striatum to the globus pallidus interna (GPi) and thereby directly modulating forebrain activity[62]. In a placebo-controlled, randomized, double-blind trial, amantadine significantly enhanced behavioral recovery in severe traumatic brain injury patients with DoC in the subacute post-injury phase[61]. The drug primarily resulted in an acceleration of the recovery as demonstrated by the washout phase when effects were compared to those that received placebo. Current guidelines recommend this as a therapy for TBI patients in the subacute phase after injury [16].

Zolpidem is thought to inhibit the GPi, which may subsequently lead to the activation of the frontal cortex and striatum, by downregulating the excess inhibition of the central thalamus[63, 64]. There is anecdotal clinical evidence for its potential to support recovery of consciousness and several physiological studies to support its potential. Clinical improvement with zolpidem correlates with increased regional metabolism on fluorodeoxyglucose positron emission tomography[186], enhanced BOLD signal of fMRI[187], and reduced burst suppression on EEG restoring thalamocortical signals[188].

Neuromodulation therapy for patients with DoC (Fig. 2):

Transcranial direct current stimulation (tDCS) applies low-level direct current (≤ 2 mA) across the cortex between two electrodes to modulate brain function[15], affecting membrane potentials and *N*-methyl-d-aspartate receptor efficiency. This may lead to long-lasting changes akin to long-term potentiation and depression [189–191]. tDCS has been found to transiently improve working memory and attention via stimulation of the left dorsolateral prefrontal cortex [192, 193] and potentially improve consciousness in patients with severe brain injury, as indicated by CRS-R scores [194].

TMS induces neural depolarization with electromagnetic pulses. Repeated TMS stimulation modifies neuronal excitability, enabling either sustained inhibition (~ 1 Hz) or activation (5–20 Hz) of neurons [15]. However, in a study assessing 20 Hz rTMS on vegetative state patients, no significant differences in CRS-R, or EEG changes were noted between real and sham stimulations [195].

Deep brain stimulation (DBS) electrodes, targeted at the central thalamic nuclei have been used to stimulate thalamo-cortical projections with a goal to improve the level of consciousness and neurological function in a few DoC patients with severe brain injury [15, 196]. This approach addresses pathophysiological alterations and cellular loss in the central thalamus [197]. In a 6-month follow-up study, this intervention improved behavioral responsiveness and cognitive functions in a patient who had been in MCS for 6 years after a TBI. Increased frequency of cognitively mediated behaviors, limb control, and oral feeding were noted with DBS activation, with statistical analysis linking these improvements to DBS use [65]. The results suggest that DBS may partially compensate for impaired regulation of arousal, typically supported by the frontal lobe. In a case series of patients with moderate-to-severe TBI, the safety and efficacy of DBS within the central lateral nucleus and the associated medial dorsal tegmental tract were demonstrated, improving executive control in patients who are in the chronic phase of recovery [66].

Low-intensity focused ultrasound pulse(LIFU) employs low-energy sound waves to modulate neural activity, offering a theoretical advantage over tDCS and rTMS by non-invasively targeting deeper neural regions, including the thalamus [15]. Clinical case reports suggest that this approach may have some promise for patients with both acute and chronic DOCs [198, 199].

Vagal nerve stimulation (VNS), available as invasive surgery with a 1–2 mA stimulator or non-invasively via ear stimulation, aims to trigger compensatory neural mechanisms via projections from the basal forebrain or brainstem through the central thalamus and hypothalamus to distal fronto-parietal and striatal regions [15]. The efficacy of VNS for DoC is still uncertain, with inconsistent findings from RCTs. A preliminary RCT on stroke and TBI patients found significant CRS-R score improvements in patients with MCS [200], while another study including HIE patients observed no substantial benefits [201]. Further research is required to elucidate the effectiveness of VNS treatments for patients with DoC.

**Ethical considerations of current practice**

Recent advancements in our understanding of DoC, coupled with the development of practice guidelines by “the American Academy of Neurology, the American Congress of Rehabilitation Medicine, and the National Institute on Disability, Independent Living, and Rehabilitation Research” [16] and “European Academy of Neurology guideline on the diagnosis of coma and other disorders of consciousness” [202], mark a significant turning point in the care of DoC patients with severe brain injuries. However, numerous challenges remain, with ethical considerations being particularly significant in clinical practice. Much of the available prognostic data is potentially biased by the self-fulfilling prophecy and early WLST [26–28]. The imprecision of current prognostic predictions for DoC, will result in variability of care with the potential for biases to influence decisions [29]. Care for patients with DoC can place significant financial and emotional strain on patients and families. Development of health systems to care for and promote recovery among patients with DoC is essential. On the other hand, advanced techniques, such as fMRI and EEG, are becoming promising tools; however, the issue of unequal access to them must be addressed to ensure that access to advanced diagnostic assessments is not restricted [203]. An important part of the advancement of recovery science is focusing on the implementation of best practices, through efforts such as provider education on the practice of communication of uncertain prognoses [204, 205]. Surrogates need to receive education to put them into a position to participate in shared decision-making for these crucial decisions [206, 207].

## Conclusion

Major advancements in understanding underlying mechanisms, developing accurate prognostications, and supporting the recovery of patients with DoC have been made but we are still at the beginning of a long road before individualized support for the recovery of these patients can be offered. The latest advancements in neuroimaging and electrophysiology technologies have brought about progress in assessing brain function in patients with DoC. Detection of CMD is emerging as a potentially important state that is still poorly understood. Enriching the knowledge of the DoC patient’s endotype will hopefully provide a more targeted and individualized approach to prognostication and treatment of the brain-injured patient with DoC. It will be essential to determine how these advancements can be implemented and benefit DoC patients across a range of clinical settings and societal systems globally [12]. The Curing Coma Campaign has identified in a number of papers the major gaps and provided a roadmap to advance the field of coma science with the goal to support the recovery of patients with DoC [12, 69, 75, 185].

## Data Availability

Not applicable.

## Abbreviations

ACCESS: Advanced classification of consciousness endotypes
ARAS: Ascending reticular activating system
BOLD: Blood oxygen level-dependent
CA: Cardiac arrest
CMD: Cognitive motor dissociation
CRS-R: Coma recovery scale-revised
CRSR-FAST: CRS-R for accelerated standardized testing
CS: Confusional state
CT: Computerized tomography
DBS: Deep brain stimulation
DMN: Default mode network
DoC: Disorders of consciousness
DTI: Diffusion tensor imaging
ECN: Executive control network
EEG: Electroencephalography
fMRI: Functional magnetic resonance imaging
GCS: Glasgow coma scale
GNW: Global neuronal workspace
GPi: Globus pallidus interna
ICH: Intracerebral hemorrhage
IIT: Integrated information theory
LIFU: Low intensity focused ultrasound pulse
MCS: Minimally conscious state
MSN: Medium spiny neurons
PCI: Perturbational complexity index
PE: Permutation entropy
PTCS: Posttraumatic confusional state
rEEG: Resting EEG
SAH: Subarachnoid hemorrhage
SN: Salience network
SVM: Support vector machine
TBI: Traumatic brain injury
tDCS: Transcranial direct current stimulation
TMS: Transcranial magnetic stimulation
UWS: Unresponsive wakefulness syndrome
VNS: Vagal nerve stimulation
VS: Vegetative state
WLST: Withdrawal of life-sustaining treatments
wSMI: Weighted symbolic mutual information

## References

[1] Giacino JT, Fins JJ, Laureys S, Schiff ND. Disorders of consciousness after acquired brain injury: the state of the science. Nat Rev Neurol. 2014;10:99–114. 24468878 10.1038/nrneurol.2013.279

[2] Brogan ME, Provencio JJ. Spectrum of catastrophic brain injury: coma and related disorders of consciousness. J Crit Care. 2014;29:679–82. 24930368 10.1016/j.jcrc.2014.04.014

[3] Giacino JT, Malone R. The vegetative and minimally conscious states. Handb Clin Neurol. 2008;90:99–111. 18631819 10.1016/S0072-9752(07)01706-X

[4] Kondziella D, Amiri M, Othman MH, Beghi E, Bodien YG, Citerio G, et al. Incidence and prevalence of coma in the UK and the USA. Brain Commun. 2022;4:fcac188. 36132425 10.1093/braincomms/fcac188 PMC9486895

[5] Demetriades D, Kuncir E, Velmahos GC, Rhee P, Alo K, Chan LS. Outcome and prognostic factors in head injuries with an admission Glasgow Coma Scale score of 3. Arch Surg. 2004;139:1066–8. 15492144 10.1001/archsurg.139.10.1066

[6] Edlow JA, Rabinstein A, Traub SJ, Wijdicks EF. Diagnosis of reversible causes of coma. Lancet. 2014;384:2064–76. 24767707 10.1016/S0140-6736(13)62184-4

[7] Kotwica Z, Jakubowski JK. Head-injured adult patients with GCS of 3 on admission—who have a chance to survive? Acta Neurochir (Wien). 1995;133:56–9. 8561037 10.1007/BF01404948

[8] Leonardi M, Giovannetti AM, Pagani M, Raggi A, Sattin D, National Consortium F, et al. Burden and needs of 487 caregivers of patients in vegetative state and in minimally conscious state: results from a national study. Brain Inj. 2012;26:1201–10. 22571687 10.3109/02699052.2012.667589

[9] Corallo F, Pria D, Di Blasi A, Bonanno L, De Cola MC, Di Cara M, et al. The effects of caregiver’s burden on dynamic structure in disorder of consciousness families: an observational study. Brain Behav. 2021;11: e2305. 34350729 10.1002/brb3.2305 PMC8413804

[10] Injury NCDPoRoPWTB. Consensus conference. Rehabilitation of persons with traumatic brain injury. NIH Consensus Development Panel on Rehabilitation of Persons With Traumatic Brain Injury. JAMA. 1999;282:974–83. 10485684

[11] Edlow BL, Claassen J, Schiff ND, Greer DM. Recovery from disorders of consciousness: mechanisms, prognosis and emerging therapies. Nat Rev Neurol. 2021;17:135–56. 33318675 10.1038/s41582-020-00428-x PMC7734616

[12] Hammond FM, Katta-Charles S, Russell MB, Zafonte RD, Claassen J, Wagner AK, et al. Research needs for prognostic modeling and trajectory analysis in patients with disorders of consciousness. Neurocrit Care. 2021;35:55–67. 34236623 10.1007/s12028-021-01289-y PMC8848858

[13] Estraneo A, Moretta P, Loreto V, Lanzillo B, Santoro L, Trojano L. Late recovery after traumatic, anoxic, or hemorrhagic long-lasting vegetative state. Neurology. 2010;75:239–45. 20554941 10.1212/WNL.0b013e3181e8e8cc

[14] Estraneo A, Moretta P, Loreto V, Santoro L, Trojano L. Clinical and neuropsychological long-term outcomes after late recovery of responsiveness: a case series. Arch Phys Med Rehabil. 2014;95:711–6. 24275063 10.1016/j.apmr.2013.11.004

[15] Thibaut A, Schiff N, Giacino J, Laureys S, Gosseries O. Therapeutic interventions in patients with prolonged disorders of consciousness. Lancet Neurol. 2019;18:600–14. 31003899 10.1016/S1474-4422(19)30031-6

[16] Giacino JT, Katz DI, Schiff ND, Whyte J, Ashman EJ, Ashwal S, et al. Practice guideline update recommendations summary: Disorders of consciousness: Report of the Guideline Development, Dissemination, and Implementation Subcommittee of the American Academy of Neurology; the American Congress of Rehabilitation Medicine; and the National Institute on Disability, Independent Living, and Rehabilitation Research. Neurology. 2018;91:450–60. 30089618 10.1212/WNL.0000000000005926 PMC6139814

[17] Kowalski RG, Hammond FM, Weintraub AH, Nakase-Richardson R, Zafonte RD, Whyte J, et al. Recovery of consciousness and functional outcome in moderate and severe traumatic brain injury. JAMA Neurol. 2021;78:548–57. 33646273 10.1001/jamaneurol.2021.0084 PMC7922241

[18] Hammond FM, Giacino JT, Nakase Richardson R, Sherer M, Zafonte RD, Whyte J, et al. Disorders of consciousness due to traumatic brain injury: functional status ten years post-injury. J Neurotrauma. 2019;36:1136–46. 30226400 10.1089/neu.2018.5954

[19] McCrea MA, Giacino JT, Barber J, Temkin NR, Nelson LD, Levin HS, et al. Functional outcomes over the first year after moderate to severe traumatic brain injury in the prospective, longitudinal TRACK-TBI study. JAMA Neurol. 2021;78:982–92. 34228047 10.1001/jamaneurol.2021.2043 PMC8261688

[20] Witsch J, Siegerink B, Nolte CH, Sprügel M, Steiner T, Endres M, et al. Prognostication after intracerebral hemorrhage: a review. Neurol Res Pract. 2021;3:22. 33934715 10.1186/s42466-021-00120-5 PMC8091769

[21] Steyerberg EW, Mushkudiani N, Perel P, Butcher I, Lu J, McHugh GS, et al. Predicting outcome after traumatic brain injury: development and international validation of prognostic scores based on admission characteristics. PLoS Med. 2008;5:e165 (**discussion e**). 18684008 10.1371/journal.pmed.0050165 PMC2494563

[22] Sivaraju A, Gilmore EJ, Wira CR, Stevens A, Rampal N, Moeller JJ, et al. Prognostication of post-cardiac arrest coma: early clinical and electroencephalographic predictors of outcome. Intensive Care Med. 2015;41:1264–72. 25940963 10.1007/s00134-015-3834-x

[23] Bae DH, Lee HY, Jung YH, Jeung KW, Lee BK, Youn CS, et al. PROLOGUE (PROgnostication using LOGistic regression model for Unselected adult cardiac arrest patients in the Early stages): development and validation of a scoring system for early prognostication in unselected adult cardiac arrest patients. Resuscitation. 2021;159:60–8. 33388366 10.1016/j.resuscitation.2020.12.022

[24] Blatter R, Gökduman B, Amacher SA, Becker C, Beck K, Gross S, et al. External validation of the PROLOGUE score to predict neurological outcome in adult patients after cardiac arrest: a prospective cohort study. Scand J Trauma Resusc Emerg Med. 2023;31:16. 37016393 10.1186/s13049-023-01081-1 PMC10074653

[25] Hwang DY, Dell CA, Sparks MJ, Watson TD, Langefeld CD, Comeau ME, et al. Clinician judgment vs formal scales for predicting intracerebral hemorrhage outcomes. Neurology. 2016;86:126–33. 26674335 10.1212/WNL.0000000000002266 PMC4731687

[26] Turgeon AF, Lauzier F, Simard JF, Scales DC, Burns KE, Moore L, et al. Mortality associated with withdrawal of life-sustaining therapy for patients with severe traumatic brain injury: a Canadian multicentre cohort study. CMAJ. 2011;183:1581–8. 21876014 10.1503/cmaj.101786 PMC3185074

[27] Alkhachroum A, Bustillo AJ, Asdaghi N, Marulanda-Londono E, Gutierrez CM, Samano D, et al. Withdrawal of life-sustaining treatment mediates mortality in patients with intracerebral hemorrhage with impaired consciousness. Stroke. 2021;52:3891–8. 34583530 10.1161/STROKEAHA.121.035233 PMC8608746

[28] Elmer J, Torres C, Aufderheide TP, Austin MA, Callaway CW, Golan E, et al. Association of early withdrawal of life-sustaining therapy for perceived neurological prognosis with mortality after cardiac arrest. Resuscitation. 2016;102:127–35. 26836944 10.1016/j.resuscitation.2016.01.016 PMC4834233

[29] Rohaut B, Claassen J. Decision making in perceived devastating brain injury: a call to explore the impact of cognitive biases. Br J Anaesth. 2018;120:5–9. 29397137 10.1016/j.bja.2017.11.007

[30] Rosenfeld JV, Maas AI, Bragge P, Morganti-Kossmann MC, Manley GT, Gruen RL. Early management of severe traumatic brain injury. Lancet. 2012;380:1088–98. 22998718 10.1016/S0140-6736(12)60864-2

[31] Gosseries O, Schnakers C, Vanhaudenhuyse A, Martial C, Aubinet C, Charland-Verville V, et al. Needs and quality of life of caregivers of patients with prolonged disorders of consciousness. Brain Sci. 2023;13:308. 36831851 10.3390/brainsci13020308 PMC9953960

[32] Makino J, Fujitani S, Twohig B, Krasnica S, Oropello J. End-of-life considerations in the ICU in Japan: ethical and legal perspectives. J Intensive Care. 2014;2:9. 25520825 10.1186/2052-0492-2-9 PMC4267582

[33] The Japanese Society of Intensive Care Medicine EC. The current situation survey about the clinical ethics in the affiliation facilities of the Japanese society of intensive care medicine councilor. J Jpn Soc Intensive Care Med. 2013;20:307–19.

[34] Yaguchi A, Truog RD, Curtis JR, Luce JM, Levy MM, Melot C, et al. International differences in end-of-life attitudes in the intensive care unit: results of a survey. Arch Intern Med. 2005;165:1970–5. 16186466 10.1001/archinte.165.17.1970

[35] Egawa S, Ader J, Shen Q, Nakagawa S, Fujimoto Y, Fujii S, et al. Long-term outcomes of patients with stroke predicted by clinicians to have no chance of meaningful recovery: a Japanese Cohort Study. Neurocrit Care. 2023;38:733–40. 36450972 10.1007/s12028-022-01644-7 PMC10227183

[36] Posner JBSC, Schiff ND, Claassen J. Plum and Posner’s: diagnosis and treatment of Stupor and Coma. 5th ed. New York: Oxford University Press; 2019.

[37] Laureys S. The neural correlate of (un)awareness: lessons from the vegetative state. Trends Cogn Sci. 2005;9:556–9. 16271507 10.1016/j.tics.2005.10.010

[38] Saper CB, Scammell TE, Lu J. Hypothalamic regulation of sleep and circadian rhythms. Nature. 2005;437:1257–63. 16251950 10.1038/nature04284

[39] Teasdale G, Jennett B. Assessment of coma and impaired consciousness. A practical scale. Lancet. 1974;2:81–4. 4136544 10.1016/s0140-6736(74)91639-0

[40] Jennett B, Plum F. Persistent vegetative state after brain damage. A syndrome in search of a name. Lancet. 1972;1:734–7. 4111204 10.1016/s0140-6736(72)90242-5

[41] Multi-Society Task Force on PVS. Medical aspects of the persistent vegetative state (1). N Engl J Med. 1994;330:1499–508. 7818633 10.1056/NEJM199405263302107

[42] Laureys S, Celesia GG, Cohadon F, Lavrijsen J, Leon-Carrion J, Sannita WG, et al. Unresponsive wakefulness syndrome: a new name for the vegetative state or apallic syndrome. BMC Med. 2010;8:68. 21040571 10.1186/1741-7015-8-68 PMC2987895

[43] Giacino JT, Ashwal S, Childs N, Cranford R, Jennett B, Katz DI, et al. The minimally conscious state: definition and diagnostic criteria. Neurology. 2002;58:349–53. 11839831 10.1212/wnl.58.3.349

[44] Thibaut A, Bodien YG, Laureys S, Giacino JT. Minimally conscious state “plus”: diagnostic criteria and relation to functional recovery. J Neurol. 2020;267:1245–54. 31773246 10.1007/s00415-019-09628-y

[45] Sherer M, Katz DI, Bodien YG, Arciniegas DB, Block C, Blum S, et al. Post-traumatic confusional state: a case definition and diagnostic criteria. Arch Phys Med Rehabil. 2020;101:2041–50. 32738198 10.1016/j.apmr.2020.06.021

[46] Symonds CP. Mental disorder following head injury: (section of psychiatry). Proc R Soc Med. 1937;30:1081–94. 19991202 10.1177/003591573703000926 PMC2076336

[47] Kwentus JA, Hart RP, Peck ET, Kornstein S. Psychiatric complications of closed head trauma. Psychosomatics. 1985;26:8–17. 3969436 10.1016/S0033-3182(85)72900-3

[48] Stuss DT, Binns MA, Carruth FG, Levine B, Brandys CE, Moulton RJ, et al. The acute period of recovery from traumatic brain injury: posttraumatic amnesia or posttraumatic confusional state? J Neurosurg. 1999;90:635–43. 10193606 10.3171/jns.1999.90.4.0635

[49] Sherer M, Nakase-Thompson R, Yablon SA, Gontkovsky ST. Multidimensional assessment of acute confusion after traumatic brain injury. Arch Phys Med Rehabil. 2005;86:896–904. 15895334 10.1016/j.apmr.2004.09.029

[50] Owen AM, Coleman MR, Boly M, Davis MH, Laureys S, Pickard JD. Detecting awareness in the vegetative state. Science. 2006;313:1402. 16959998 10.1126/science.1130197

[51] Schiff ND. Cognitive motor dissociation following severe brain injuries. JAMA Neurol. 2015;72:1413–5. 26502348 10.1001/jamaneurol.2015.2899

[52] Edlow BL, Chatelle C, Spencer CA, Chu CJ, Bodien YG, O’Connor KL, et al. Early detection of consciousness in patients with acute severe traumatic brain injury. Brain. 2017;140:2399–414. 29050383 10.1093/brain/awx176 PMC6059097

[53] Wijdicks EFM. The practice of emergency and critical care neurology. Oxford: Oxford University Press; 2016. p. 112.

[54] Zeman A. Persistent vegetative state. Lancet. 1997;350:795–9. 9298013 10.1016/S0140-6736(97)06447-7

[55] Dehaene S, Kerszberg M, Changeux JP. A neuronal model of a global workspace in effortful cognitive tasks. Proc Natl Acad Sci USA. 1998;95:14529–34. 9826734 10.1073/pnas.95.24.14529 PMC24407

[56] Desmedt JE. P300 in serial tasks: an essential post-decision closure mechanism. Prog Brain Res. 1980;54:682–6. 7220985 10.1016/S0079-6123(08)61690-8

[57] Kestens K, Van Yper L, Degeest S, Keppler H. The P300 auditory evoked potential: a physiological measure of the engagement of cognitive systems contributing to listening effort? Ear Hear. 2023;44:1389–403. 37287098 10.1097/AUD.0000000000001381

[58] Tononi G, Boly M, Massimini M, Koch C. Integrated information theory: from consciousness to its physical substrate. Nat Rev Neurosci. 2016;17:450–61. 27225071 10.1038/nrn.2016.44

[59] Casali AG, Gosseries O, Rosanova M, Boly M, Sarasso S, Casali KR, et al. A theoretically based index of consciousness independent of sensory processing and behavior. Sci Transl Med. 2013;5:198ra05. 10.1126/scitranslmed.3006294 23946194

[60] Schiff ND. Recovery of consciousness after brain injury: a mesocircuit hypothesis. Trends Neurosci. 2010;33:1–9. 19954851 10.1016/j.tins.2009.11.002 PMC2931585

[61] Giacino JT, Whyte J, Bagiella E, Kalmar K, Childs N, Khademi A, et al. Placebo-controlled trial of amantadine for severe traumatic brain injury. N Engl J Med. 2012;366:819–26. 22375973 10.1056/NEJMoa1102609

[62] Schnakers C, Hustinx R, Vandewalle G, Majerus S, Moonen G, Boly M, et al. Measuring the effect of amantadine in chronic anoxic minimally conscious state. J Neurol Neurosurg Psychiatry. 2008;79:225–7. 18202217 10.1136/jnnp.2007.124099

[63] Fridman EA, Schiff ND. Neuromodulation of the conscious state following severe brain injuries. Curr Opin Neurobiol. 2014;29:172–7. 25285395 10.1016/j.conb.2014.09.008 PMC6519077

[64] Williams ST, Conte MM, Goldfine AM, Noirhomme Q, Gosseries O, Thonnard M, et al. Common resting brain dynamics indicate a possible mechanism underlying zolpidem response in severe brain injury. Elife. 2013;2: e01157. 24252875 10.7554/eLife.01157 PMC3833342

[65] Schiff ND, Giacino JT, Kalmar K, Victor JD, Baker K, Gerber M, et al. Behavioural improvements with thalamic stimulation after severe traumatic brain injury. Nature. 2007;448:600–3. 17671503 10.1038/nature06041

[66] Schiff ND, Giacino JT, Butson CR, Choi EY, Baker JL, O’Sullivan KP, et al. Thalamic deep brain stimulation in traumatic brain injury: a phase 1, randomized feasibility study. Nat Med. 2023;29:3162–74. 38049620 10.1038/s41591-023-02638-4 PMC11087147

[67] Logi F, Pasqualetti P, Tomaiuolo F. Predict recovery of consciousness in post-acute severe brain injury: the role of EEG reactivity. Brain Inj. 2011;25:972–9. 21745174 10.3109/02699052.2011.589795

[68] Attia J, Cook DJ. Prognosis in anoxic and traumatic coma. Crit Care Clin. 1998;14:497–511. 9700444 10.1016/s0749-0704(05)70013-0

[69] Claassen J, Akbari Y, Alexander S, Bader MK, Bell K, Bleck TP, et al. Proceedings of the first curing coma campaign NIH symposium: challenging the future of research for coma and disorders of consciousness. Neurocrit Care. 2021;35:4–23. 34236619 10.1007/s12028-021-01260-x PMC8264966

[70] Quinn T, Moskowitz J, Khan MW, Shutter L, Goldberg R, Col N, et al. What families need and physicians deliver: contrasting communication preferences between surrogate decision-makers and physicians during outcome prognostication in critically ill TBI patients. Neurocrit Care. 2017;27:154–62. 28685395 10.1007/s12028-017-0427-2 PMC5693603

[71] Christakis NA, Iwashyna TJ. Attitude and self-reported practice regarding prognostication in a national sample of internists. Arch Intern Med. 1998;158:2389–95. 9827791 10.1001/archinte.158.21.2389

[72] Geurts M, Macleod MR, van Thiel GJ, van Gijn J, Kappelle LJ, van der Worp HB. End-of-life decisions in patients with severe acute brain injury. Lancet Neurol. 2014;13:515–24. 24675048 10.1016/S1474-4422(14)70030-4

[73] Qiu H, Zador Z, Lannon M, Farrokhyar F, Duda T, Sharma S. Identification of clinically relevant patient endotypes in traumatic brain injury using latent class analysis. Sci Rep. 2024;14:1294. 38221527 10.1038/s41598-024-51474-0 PMC10788338

[74] Azad TD, Shah PP, Kim HB, Stevens RD. Endotypes and the path to precision in moderate and severe traumatic brain injury. Neurocrit Care. 2022;37:259–66. 35314969 10.1007/s12028-022-01475-6

[75] Kondziella D, Menon DK, Helbok R, Naccache L, Othman MH, Rass V, et al. A precision medicine framework for classifying patients with disorders of consciousness: advanced classification of consciousness endotypes (ACCESS). Neurocrit Care. 2021;35:27–36. 34236621 10.1007/s12028-021-01246-9

[76] Giacino JT, Kalmar K, Whyte J. The JFK coma recovery scale-revised: measurement characteristics and diagnostic utility. Arch Phys Med Rehabil. 2004;85:2020–9. 15605342 10.1016/j.apmr.2004.02.033

[77] Bodien YG, Carlowicz CA, Chatelle C, Giacino JT. Sensitivity and specificity of the coma recovery scale-revised total score in detection of conscious awareness. Arch Phys Med Rehabil. 2016;97:490-2.e1. 26342571 10.1016/j.apmr.2015.08.422 PMC5018674

[78] Boltzmann M, Schmidt SB, Gutenbrunner C, Krauss JK, Stangel M, Höglinger GU, et al. The influence of the CRS-R score on functional outcome in patients with severe brain injury receiving early rehabilitation. BMC Neurol. 2021;21:44. 33514337 10.1186/s12883-021-02063-5 PMC7847163

[79] Bodien YG, Vora I, Barra A, Chiang K, Chatelle C, Goostrey K, et al. Feasibility and validity of the coma recovery scale-revised for accelerated standardized testing: a practical assessment tool for detecting consciousness in the intensive care unit. Ann Neurol. 2023;94:919–24. 37488068 10.1002/ana.26740 PMC10701693

[80] Saatman KE, Duhaime AC, Bullock R, Maas AI, Valadka A, Manley GT, et al. Classification of traumatic brain injury for targeted therapies. J Neurotrauma. 2008;25:719–38. 18627252 10.1089/neu.2008.0586 PMC2721779

[81] Wu O, Sorensen AG, Benner T, Singhal AB, Furie KL, Greer DM. Comatose patients with cardiac arrest: predicting clinical outcome with diffusion-weighted MR imaging. Radiology. 2009;252:173–81. 19420318 10.1148/radiol.2521081232 PMC2702469

[82] Wijman CA, Mlynash M, Caulfield AF, Hsia AW, Eyngorn I, Bammer R, et al. Prognostic value of brain diffusion-weighted imaging after cardiac arrest. Ann Neurol. 2009;65:394–402. 19399889 10.1002/ana.21632 PMC2677115

[83] Greer DM, Scripko PD, Wu O, Edlow BL, Bartscher J, Sims JR, et al. Hippocampal magnetic resonance imaging abnormalities in cardiac arrest are associated with poor outcome. J Stroke Cerebrovasc Dis. 2013;22:899–905. 22995378 10.1016/j.jstrokecerebrovasdis.2012.08.006

[84] Tong KA, Ashwal S, Holshouser BA, Nickerson JP, Wall CJ, Shutter LA, et al. Diffuse axonal injury in children: clinical correlation with hemorrhagic lesions. Ann Neurol. 2004;56:36–50. 15236400 10.1002/ana.20123

[85] Yanagawa Y, Tsushima Y, Tokumaru A, Un-no Y, Sakamoto T, Okada Y, et al. A quantitative analysis of head injury using T2*-weighted gradient-echo imaging. J Trauma. 2000;49:272–7. 10963538 10.1097/00005373-200008000-00013

[86] Griffin AD, Turtzo LC, Parikh GY, Tolpygo A, Lodato Z, Moses AD, et al. Traumatic microbleeds suggest vascular injury and predict disability in traumatic brain injury. Brain. 2019;142:3550–64. 31608359 10.1093/brain/awz290 PMC6821371

[87] Izzy S, Mazwi NL, Martinez S, Spencer CA, Klein JP, Parikh G, et al. Revisiting grade 3 diffuse axonal injury: not all brainstem microbleeds are prognostically equal. Neurocrit Care. 2017;27:199–207. 28477152 10.1007/s12028-017-0399-2 PMC5877823

[88] Gentry LR, Godersky JC, Thompson B, Dunn VD. Prospective comparative study of intermediate-field MR and CT in the evaluation of closed head trauma. AJR Am J Roentgenol. 1988;150:673–82. 3257625 10.2214/ajr.150.3.673

[89] Skandsen T, Kvistad KA, Solheim O, Strand IH, Folvik M, Vik A. Prevalence and impact of diffuse axonal injury in patients with moderate and severe head injury: a cohort study of early magnetic resonance imaging findings and 1-year outcome. J Neurosurg. 2010;113:556–63. 19852541 10.3171/2009.9.JNS09626

[90] Edlow BL, Giacino JT, Hirschberg RE, Gerrard J, Wu O, Hochberg LR. Unexpected recovery of function after severe traumatic brain injury: the limits of early neuroimaging-based outcome prediction. Neurocrit Care. 2013;19:364–75. 23860665 10.1007/s12028-013-9870-x PMC3902071

[91] Muccio CF, De Simone M, Esposito G, De Blasio E, Vittori C, Cerase A. Reversible post-traumatic bilateral extensive restricted diffusion of the brain. A case study and review of the literature. Brain Inj. 2009;23:466–72. 19408169 10.1080/02699050902841912

[92] Edlow BL, Haynes RL, Takahashi E, Klein JP, Cummings P, Benner T, et al. Disconnection of the ascending arousal system in traumatic coma. J Neuropathol Exp Neurol. 2013;72:505–23. 23656993 10.1097/NEN.0b013e3182945bf6 PMC3761353

[93] Galanaud D, Perlbarg V, Gupta R, Stevens RD, Sanchez P, Tollard E, et al. Assessment of white matter injury and outcome in severe brain trauma: a prospective multicenter cohort. Anesthesiology. 2012;117:1300–10. 23135261 10.1097/ALN.0b013e3182755558

[94] Velly L, Perlbarg V, Boulier T, Adam N, Delphine S, Luyt CE, et al. Use of brain diffusion tensor imaging for the prediction of long-term neurological outcomes in patients after cardiac arrest: a multicentre, international, prospective, observational, cohort study. Lancet Neurol. 2018;17:317–26. 29500154 10.1016/S1474-4422(18)30027-9

[95] Puybasset L, Perlbarg V, Unrug J, Cassereau D, Galanaud D, Torkomian G, et al. Prognostic value of global deep white matter DTI metrics for 1-year outcome prediction in ICU traumatic brain injury patients: an MRI-COMA and CENTER-TBI combined study. Intensive Care Med. 2022;48:201–12. 34904191 10.1007/s00134-021-06583-z

[96] Yuh EL, Cooper SR, Mukherjee P, Yue JK, Lingsma HF, Gordon WA, et al. Diffusion tensor imaging for outcome prediction in mild traumatic brain injury: a TRACK-TBI study. J Neurotrauma. 2014;31:1457–77. 24742275 10.1089/neu.2013.3171 PMC4144386

[97] Palacios EM, Yuh EL, Mac Donald CL, Bourla I, Wren-Jarvis J, Sun X, et al. Diffusion tensor imaging reveals elevated diffusivity of white matter microstructure that is independently associated with long-term outcome after mild traumatic brain injury: a traCK-TBI study. J Neurotrauma. 2022;39:1318–28. 35579949 10.1089/neu.2021.0408 PMC9529303

[98] Maffei C, Gilmore N, Snider SB, Foulkes AS, Bodien YG, Yendiki A, et al. Automated detection of axonal damage along white matter tracts in acute severe traumatic brain injury. Neuroimage Clin. 2023;37: 103294. 36529035 10.1016/j.nicl.2022.103294 PMC9792957

[99] Wang JY, Bakhadirov K, Abdi H, Devous MD, Marquez de la Plata CD, Moore C, et al. Longitudinal changes of structural connectivity in traumatic axonal injury. Neurology. 2011;77:818–26. 21813787 10.1212/WNL.0b013e31822c61d7 PMC3162636

[100] Edlow BL, Copen WA, Izzy S, Bakhadirov K, van der Kouwe A, Glenn MB, et al. Diffusion tensor imaging in acute-to-subacute traumatic brain injury: a longitudinal analysis. BMC Neurol. 2016;16:2. 26754948 10.1186/s12883-015-0525-8 PMC4707723

[101] Warner MA, MarquezdelaPlata C, Spence J, Wang JY, Harper C, Moore C, et al. Assessing spatial relationships between axonal integrity, regional brain volumes, and neuropsychological outcomes after traumatic axonal injury. J Neurotrauma. 2010;27:2121–30. 20874032 10.1089/neu.2010.1429 PMC2996819

[102] Snider SB, Bodien YG, Bianciardi M, Brown EN, Wu O, Edlow BL. Disruption of the ascending arousal network in acute traumatic disorders of consciousness. Neurology. 2019;93:e1281–7. 31484715 10.1212/WNL.0000000000008163 PMC7011864

[103] Edlow BL, Barra ME, Zhou DW, Foulkes AS, Snider SB, Threlkeld ZD, et al. Personalized connectome mapping to guide targeted therapy and promote recovery of consciousness in the intensive care unit. Neurocrit Care. 2020;33:364–75. 32794142 10.1007/s12028-020-01062-7 PMC8336723

[104] Xu LB, Hampton S, Fischer D. Neuroimaging in disorders of consciousness and recovery. Phys Med Rehabil Clin N Am. 2024;35:51–64. 37993193 10.1016/j.pmr.2023.06.017 PMC12519885

[105] Farg H, Elnakib A, Gebreil A, Alksas A, van Bogaert E, Mahmoud A, et al. Diagnostic value of PET imaging in clinically unresponsive patients. Br J Radiol. 2024;97:283–91. 38308033 10.1093/bjr/tqad040

[106] Fischer D, Newcombe V, Fernandez-Espejo D, Snider SB. Applications of advanced MRI to disorders of consciousness. Semin Neurol. 2022;42:325–34. 35790201 10.1055/a-1892-1894 PMC12703101

[107] Sanz LRD, Thibaut A, Edlow BL, Laureys S, Gosseries O. Update on neuroimaging in disorders of consciousness. Curr Opin Neurol. 2021;34:488–96. 34054109 10.1097/WCO.0000000000000951 PMC8938964

[108] Jordan KG. Emergency EEG and continuous EEG monitoring in acute ischemic stroke. J Clin Neurophysiol. 2004;21:341–52. 15592008

[109] Sharbrough FW, Messick JM Jr, Sundt TM Jr. Correlation of continuous electroencephalograms with cerebral blood flow measurements during carotid endarterectomy. Stroke. 1973;4:674–83. 4723697 10.1161/01.str.4.4.674

[110] Rosenthal ES, Biswal S, Zafar SF, O’Connor KL, Bechek S, Shenoy AV, et al. Continuous electroencephalography predicts delayed cerebral ischemia after subarachnoid hemorrhage: a prospective study of diagnostic accuracy. Ann Neurol. 2018;83:958–69. 29659050 10.1002/ana.25232 PMC6021198

[111] Claassen J, Hirsch LJ, Kreiter KT, Du EY, Connolly ES, Emerson RG, et al. Quantitative continuous EEG for detecting delayed cerebral ischemia in patients with poor-grade subarachnoid hemorrhage. Clin Neurophysiol. 2004;115:2699–710. 15546778 10.1016/j.clinph.2004.06.017

[112] Hwang J, Cho SM, Ritzl EK. Recent applications of quantitative electroencephalography in adult intensive care units: a comprehensive review. J Neurol. 2022;269:6290–309. 35986096 10.1007/s00415-022-11337-y PMC9633458

[113] Wutzl B, Golaszewski SM, Leibnitz K, Langthaler PB, Kunz AB, Leis S, et al. Narrative review: quantitative EEG in disorders of consciousness. Brain Sci. 2021;11. 10.3390/brainsci11060697 PMC8228474 34070647

[114] Beniczky S, Schomer DL. Electroencephalography: basic biophysical and technological aspects important for clinical applications. Epileptic Disord. 2020;22:697–715. 33270023 10.1684/epd.2020.1217

[115] Curley WH, Bodien YG, Zhou DW, Conte MM, Foulkes AS, Giacino JT, et al. Electrophysiological correlates of thalamocortical function in acute severe traumatic brain injury. Cortex. 2022;152:136–52. 35569326 10.1016/j.cortex.2022.04.007 PMC9759728

[116] Schiff ND, Nauvel T, Victor JD. Large-scale brain dynamics in disorders of consciousness. Curr Opin Neurobiol. 2014;25:7–14. 24709594 10.1016/j.conb.2013.10.007 PMC3980494

[117] Forgacs PB, Devinsky O, Schiff ND. Independent functional outcomes after prolonged coma following cardiac arrest: a mechanistic hypothesis. Ann Neurol. 2020;87:618–32. 31994749 10.1002/ana.25690 PMC7393600

[118] Forgacs PB, Allen BB, Wu X, Gerber LM, Boddu S, Fakhar M, et al. Corticothalamic connectivity in aneurysmal subarachnoid hemorrhage: relationship with disordered consciousness and clinical outcomes. Neurocrit Care. 2022;36:760–71. 34669180 10.1007/s12028-021-01354-6

[119] Bassani T, Nievola JC. Brain-computer interface using wavelet transformation and naive bayes classifier. Adv Exp Med Biol. 2010;657:147–65. 20020346 10.1007/978-0-387-79100-5_8

[120] Holler Y, Bergmann J, Thomschewski A, Kronbichler M, Holler P, Crone JS, et al. Comparison of EEG-features and classification methods for motor imagery in patients with disorders of consciousness. PLoS ONE. 2013;8: e80479. 24282545 10.1371/journal.pone.0080479 PMC3839976

[121] Imperatori LS, Betta M, Cecchetti L, Canales-Johnson A, Ricciardi E, Siclari F, et al. EEG functional connectivity metrics wPLI and wSMI account for distinct types of brain functional interactions. Sci Rep. 2019;9:8894. 31222021 10.1038/s41598-019-45289-7 PMC6586889

[122] Sitt JD, King JR, El Karoui I, Rohaut B, Faugeras F, Gramfort A, et al. Large scale screening of neural signatures of consciousness in patients in a vegetative or minimally conscious state. Brain. 2014;137:2258–70. 24919971 10.1093/brain/awu141 PMC4610185

[123] Lehembre R, Marie-Aurelie B, Vanhaudenhuyse A, Chatelle C, Cologan V, Leclercq Y, et al. Resting-state EEG study of comatose patients: a connectivity and frequency analysis to find differences between vegetative and minimally conscious states. Funct Neurol. 2012;27:41–7. 22687166 PMC3812750

[124] King JR, Sitt JD, Faugeras F, Rohaut B, El Karoui I, Cohen L, et al. Information sharing in the brain indexes consciousness in noncommunicative patients. Curr Biol. 2013;23:1914–9. 24076243 10.1016/j.cub.2013.07.075 PMC5635964

[125] Staniek M, Lehnertz K. Symbolic transfer entropy. Phys Rev Lett. 2008;100: 158101. 18518155 10.1103/PhysRevLett.100.158101

[126] Olofsen E, Sleigh JW, Dahan A. Permutation entropy of the electroencephalogram: a measure of anaesthetic drug effect. Br J Anaesth. 2008;101:810–21. 18852113 10.1093/bja/aen290

[127] Jordan D, Stockmanns G, Kochs EF, Pilge S, Schneider G. Electroencephalographic order pattern analysis for the separation of consciousness and unconsciousness: an analysis of approximate entropy, permutation entropy, recurrence rate, and phase coupling of order recurrence plots. Anesthesiology. 2008;109:1014–22. 19034098 10.1097/ALN.0b013e31818d6c55

[128] Thul A, Lechinger J, Donis J, Michitsch G, Pichler G, Kochs EF, et al. EEG entropy measures indicate decrease of cortical information processing in disorders of consciousness. Clin Neurophysiol. 2016;127:1419–27. 26480834 10.1016/j.clinph.2015.07.039

[129] Bandt C, Pompe B. Permutation entropy: a natural complexity measure for time series. Phys Rev Lett. 2002;88: 174102. 12005759 10.1103/PhysRevLett.88.174102

[130] Bauerschmidt A, Eliseyev A, Doyle KW, Velasquez A, Egbebike J, Chiu W, et al. Predicting early recovery of consciousness after cardiac arrest supported by quantitative electroencephalography. Resuscitation. 2021;165:130–7. 34166746 10.1016/j.resuscitation.2021.06.008 PMC10008439

[131] Wielek T, Lechinger J, Wislowska M, Blume C, Ott P, Wegenkittl S, et al. Sleep in patients with disorders of consciousness characterized by means of machine learning. PLoS ONE. 2018;13: e0190458. 29293607 10.1371/journal.pone.0190458 PMC5749793

[132] Hall SD, Yamawaki N, Fisher AE, Clauss RP, Woodhall GL, Stanford IM. GABA(A) alpha-1 subunit mediated desynchronization of elevated low frequency oscillations alleviates specific dysfunction in stroke—a case report. Clin Neurophysiol. 2010;121:549–55. 20097125 10.1016/j.clinph.2009.11.084

[133] Boly M, Moran R, Murphy M, Boveroux P, Bruno MA, Noirhomme Q, et al. Connectivity changes underlying spectral EEG changes during propofol-induced loss of consciousness. J Neurosci. 2012;32:7082–90. 22593076 10.1523/JNEUROSCI.3769-11.2012 PMC3366913

[134] Breshears JD, Roland JL, Sharma M, Gaona CM, Freudenburg ZV, Tempelhoff R, et al. Stable and dynamic cortical electrophysiology of induction and emergence with propofol anesthesia. Proc Natl Acad Sci USA. 2010;107:21170–5. 21078987 10.1073/pnas.1011949107 PMC3000270

[135] Cimenser A, Purdon PL, Pierce ET, Walsh JL, Salazar-Gomez AF, Harrell PG, et al. Tracking brain states under general anesthesia by using global coherence analysis. Proc Natl Acad Sci USA. 2011;108:8832–7. 21555565 10.1073/pnas.1017041108 PMC3102391

[136] Purdon PL, Pierce ET, Mukamel EA, Prerau MJ, Walsh JL, Wong KF, et al. Electroencephalogram signatures of loss and recovery of consciousness from propofol. Proc Natl Acad Sci USA. 2013;110:E1142–51. 23487781 10.1073/pnas.1221180110 PMC3607036

[137] Frohlich J, Toker D, Monti MM. Consciousness among delta waves: a paradox? Brain. 2021;144:2257–77. 33693596 10.1093/brain/awab095

[138] Emmady PD, Anilkumar AC. EEG Abnormal Waveforms. StatPearls. Treasure Island (FL): StatPearls Publishing. Copyright^©^ 2024, StatPearls Publishing LLC.; 2024. 32491587

[139] Andraus ME, Alves-Leon SV. Non-epileptiform EEG abnormalities: an overview. Arq Neuropsiquiatr. 2011;69:829–35. 22042190 10.1590/s0004-282x2011000600020

[140] Britton JW, Frey LC, Hopp JL, Korb P, Koubeissi MZ, Lievens WE, et al. In: St. Louis EK, Frey LC editors. Electroencephalography (EEG): An Introductory Text and Atlas of Normal and Abnormal Findings in Adults, Children, and Infants. Chicago: American Epilepsy Society. Copyright ©2016 by American Epilepsy Society.; 2016. 27748095

[141] Walter WG. The location of cerebral tumours by electro-encephalography. The Lancet. 1936;228:305–8.

[142] Schaul N. The fundamental neural mechanisms of electroencephalography. Electroencephalogr Clin Neurophysiol. 1998;106:101–7. 9741769 10.1016/s0013-4694(97)00111-9

[143] Sanders RD, Mostert N, Lindroth H, Tononi G, Sleigh J. Is consciousness frontal? Two perioperative case reports that challenge that concept. Br J Anaesth. 2018;121:330–2. 29935590 10.1016/j.bja.2018.01.010 PMC6617961

[144] Gaskell AL, Hight DF, Winders J, Tran G, Defresne A, Bonhomme V, et al. Frontal alpha-delta EEG does not preclude volitional response during anaesthesia: prospective cohort study of the isolated forearm technique. Br J Anaesth. 2017;119:664–73. 29121278 10.1093/bja/aex170

[145] Palanca BJA, Wildes TS, Ju YS, Ching S, Avidan MS. Electroencephalography and delirium in the postoperative period. Br J Anaesth. 2017;119:294–307. 28854540 10.1093/bja/aew475 PMC6172974

[146] Supp GG, Siegel M, Hipp JF, Engel AK. Cortical hypersynchrony predicts breakdown of sensory processing during loss of consciousness. Curr Biol. 2011;21:1988–93. 22100063 10.1016/j.cub.2011.10.017

[147] Gosseries O, Schnakers C, Ledoux D, Vanhaudenhuyse A, Bruno MA, Demertzi A, et al. Automated EEG entropy measurements in coma, vegetative state/unresponsive wakefulness syndrome and minimally conscious state. Funct Neurol. 2011;26:25–30. 21693085 PMC3814509

[148] Brown EN, Lydic R, Schiff ND. General anesthesia, sleep, and coma. N Engl J Med. 2010;363:2638–50. 21190458 10.1056/NEJMra0808281 PMC3162622

[149] Mashour GA, Pal D, Brown EN. Prefrontal cortex as a key node in arousal circuitry. Trends Neurosci. 2022;45:722–32. 35995629 10.1016/j.tins.2022.07.002 PMC9492635

[150] Purdon PL, Sampson A, Pavone KJ, Brown EN. Clinical electroencephalography for anesthesiologists: Part I: background and basic signatures. Anesthesiology. 2015;123:937–60. 26275092 10.1097/ALN.0000000000000841 PMC4573341

[151] Reshef ER, Schiff ND, Brown EN. A neurologic examination for anesthesiologists: assessing arousal level during induction, maintenance, and emergence. Anesthesiology. 2019;130:462–71. 30664547 10.1097/ALN.0000000000002559

[152] Akeju O, Brown EN. Neural oscillations demonstrate that general anesthesia and sedative states are neurophysiologically distinct from sleep. Curr Opin Neurobiol. 2017;44:178–85. 28544930 10.1016/j.conb.2017.04.011 PMC5520989

[153] Claassen J, Velazquez A, Meyers E, Witsch J, Falo MC, Park S, et al. Bedside quantitative electroencephalography improves assessment of consciousness in comatose subarachnoid hemorrhage patients. Ann Neurol. 2016;80:541–53. 27472071 10.1002/ana.24752 PMC5042849

[154] Purdon PL, Pavone KJ, Akeju O, Smith AC, Sampson AL, Lee J, et al. The Ageing Brain: age-dependent changes in the electroencephalogram during propofol and sevoflurane general anaesthesia. Br J Anaesth. 2015;115(Suppl 1):i46–57. 26174300 10.1093/bja/aev213 PMC4501918

[155] Cornelissen L, Kim SE, Purdon PL, Brown EN, Berde CB. Age-dependent electroencephalogram (EEG) patterns during sevoflurane general anesthesia in infants. Elife. 2015;4: e06513. 26102526 10.7554/eLife.06513 PMC4502759

[156] Lewis LD, Weiner VS, Mukamel EA, Donoghue JA, Eskandar EN, Madsen JR, et al. Rapid fragmentation of neuronal networks at the onset of propofol-induced unconsciousness. Proc Natl Acad Sci USA. 2012;109:E3377–86. 23129622 10.1073/pnas.1210907109 PMC3523833

[157] Huupponen E, Maksimow A, Lapinlampi P, Särkelä M, Saastamoinen A, Snapir A, et al. Electroencephalogram spindle activity during dexmedetomidine sedation and physiological sleep. Acta Anaesthesiol Scand. 2008;52:289–94. 18005372 10.1111/j.1399-6576.2007.01537.x

[158] Engemann DA, Raimondo F, King JR, Rohaut B, Louppe G, Faugeras F, et al. Robust EEG-based cross-site and cross-protocol classification of states of consciousness. Brain. 2018;141:3179–92. 30285102 10.1093/brain/awy251

[159] Shirer WR, Ryali S, Rykhlevskaia E, Menon V, Greicius MD. Decoding subject-driven cognitive states with whole-brain connectivity patterns. Cereb Cortex. 2012;22:158–65. 21616982 10.1093/cercor/bhr099 PMC3236795

[160] Pugin D, Hofmeister J, Gasche Y, Vulliemoz S, Lövblad KO, Ville DV, et al. Resting-state brain activity for early prediction outcome in postanoxic patients in a coma with indeterminate clinical prognosis. AJNR Am J Neuroradiol. 2020;41:1022–30. 32439642 10.3174/ajnr.A6572 PMC7342736

[161] Fox MD, Raichle ME. Spontaneous fluctuations in brain activity observed with functional magnetic resonance imaging. Nat Rev Neurosci. 2007;8:700–11. 17704812 10.1038/nrn2201

[162] Schimmelpfennig J, Topczewski J, Zajkowski W, Jankowiak-Siuda K. The role of the salience network in cognitive and affective deficits. Front Hum Neurosci. 2023;17:1133367. 37020493 10.3389/fnhum.2023.1133367 PMC10067884

[163] Bodien YG, Chatelle C, Edlow BL. Functional networks in disorders of consciousness. Semin Neurol. 2017;37:485–502. 29207410 10.1055/s-0037-1607310 PMC5884076

[164] Seeley WW, Menon V, Schatzberg AF, Keller J, Glover GH, Kenna H, et al. Dissociable intrinsic connectivity networks for salience processing and executive control. J Neurosci. 2007;27:2349–56. 17329432 10.1523/JNEUROSCI.5587-06.2007 PMC2680293

[165] Davey CG, Pujol J, Harrison BJ. Mapping the self in the brain’s default mode network. Neuroimage. 2016;132:390–7. 26892855 10.1016/j.neuroimage.2016.02.022

[166] Threlkeld ZD, Bodien YG, Rosenthal ES, Giacino JT, Nieto-Castanon A, Wu O, et al. Functional networks reemerge during recovery of consciousness after acute severe traumatic brain injury. Cortex. 2018;106:299–308. 29871771 10.1016/j.cortex.2018.05.004 PMC6120794

[167] Koenig MA, Holt JL, Ernst T, Buchthal SD, Nakagawa K, Stenger VA, et al. MRI default mode network connectivity is associated with functional outcome after cardiopulmonary arrest. Neurocrit Care. 2014;20:348–57. 24464830 10.1007/s12028-014-9953-3 PMC4136809

[168] Norton L, Hutchison RM, Young GB, Lee DH, Sharpe MD, Mirsattari SM. Disruptions of functional connectivity in the default mode network of comatose patients. Neurology. 2012;78:175–81. 22218274 10.1212/WNL.0b013e31823fcd61

[169] Menon V, Uddin LQ. Saliency, switching, attention and control: a network model of insula function. Brain Struct Funct. 2010;214:655–67. 20512370 10.1007/s00429-010-0262-0 PMC2899886

[170] Sridharan D, Levitin DJ, Menon V. A critical role for the right fronto-insular cortex in switching between central-executive and default-mode networks. Proc Natl Acad Sci USA. 2008;105:12569–74. 18723676 10.1073/pnas.0800005105 PMC2527952

[171] Mikell CB, Banks GP, Frey HP, Youngerman BE, Nelp TB, Karas PJ, et al. Frontal networks associated with command following after hemorrhagic stroke. Stroke. 2015;46:49–57. 25492905 10.1161/STROKEAHA.114.007645

[172] Amiri M, Fisher PM, Raimondo F, Sidaros A, Cacic Hribljan M, Othman MH, et al. Multimodal prediction of residual consciousness in the intensive care unit: the CONNECT-ME study. Brain. 2023;146:50–64. 36097353 10.1093/brain/awac335 PMC9825454

[173] Curley WH, Forgacs PB, Voss HU, Conte MM, Schiff ND. Characterization of EEG signals revealing covert cognition in the injured brain. Brain. 2018;141:1404–21. 29562312 10.1093/brain/awy070 PMC5917770

[174] Goldfine AM, Victor JD, Conte MM, Bardin JC, Schiff ND. Determination of awareness in patients with severe brain injury using EEG power spectral analysis. Clin Neurophysiol. 2011;122:2157–68. 21514214 10.1016/j.clinph.2011.03.022 PMC3162107

[175] Claassen J, Doyle K, Matory A, Couch C, Burger KM, Velazquez A, et al. Detection of brain activation in unresponsive patients with acute brain injury. N Engl J Med. 2019;380:2497–505. 31242361 10.1056/NEJMoa1812757

[176] Bodien YG, Allanson J, Cardone P, Bonhomme A, Carmona J, Chatelle C, et al. Cognitive motor dissociation in disorders of consciousness. N Engl J Med. 2024;391:598–608. 39141852 10.1056/NEJMoa2400645 PMC7617195

[177] Egbebike J, Shen Q, Doyle K, Der-Nigoghossian CA, Panicker L, Gonzales IJ, et al. Cognitive-motor dissociation and time to functional recovery in patients with acute brain injury in the USA: a prospective observational cohort study. Lancet Neurol. 2022;21:704–13. 35841909 10.1016/S1474-4422(22)00212-5 PMC9476646

[178] Kondziella D, Friberg CK, Frokjaer VG, Fabricius M, Moller K. Preserved consciousness in vegetative and minimal conscious states: systematic review and meta-analysis. J Neurol Neurosurg Psychiatry. 2016;87:485–92. 26139551 10.1136/jnnp-2015-310958

[179] Claassen J, Kondziella D, Alkhachroum A, Diringer M, Edlow BL, Fins JJ, et al. Cognitive motor dissociation: gap analysis and future directions. Neurocrit Care. 2024;40:81–98. 37349602 10.1007/s12028-023-01769-3 PMC12517403

[180] Fernández-Espejo D, Rossit S, Owen AM. A thalamocortical mechanism for the absence of overt motor behavior in covertly aware patients. JAMA Neurol. 2015;72:1442–50. 26501399 10.1001/jamaneurol.2015.2614

[181] Franzova E, Shen Q, Doyle K, Chen JM, Egbebike J, Vrosgou A, et al. Injury patterns associated with cognitive motor dissociation. Brain. 2023;146:4645. 37574216 10.1093/brain/awad197 PMC10629765

[182] Kundu B, Brock AA, Englot DJ, Butson CR, Rolston JD. Deep brain stimulation for the treatment of disorders of consciousness and cognition in traumatic brain injury patients: a review. Neurosurg Focus. 2018;45:E14. 30064315 10.3171/2018.5.FOCUS18168 PMC6193266

[183] Shimia M, Iranmehr A, Valizadeh A, Mirzaei F, Namvar M, Rafiei E, et al. A placebo-controlled randomized clinical trial of amantadine hydrochloride for evaluating the functional improvement of patients following severe acute traumatic brain injury. J Neurosurg Sci. 2023;67:598–604. 34114429 10.23736/S0390-5616.21.05266-8

[184] Passman JN, Cleri NA, Saadon JR, Naddaf N, Gilotra K, Swarna S, et al. In-hospital amantadine does not improve outcomes after severe traumatic brain injury: an 11-year propensity-matched retrospective analysis. World Neurosurg. 2023;177:e277. 10.1016/j.wneu.2023.06.034 37331473

[185] Edlow BL, Sanz LRD, Polizzotto L, Pouratian N, Rolston JD, Snider SB, et al. Therapies to restore consciousness in patients with severe brain injuries: a gap analysis and future directions. Neurocrit Care. 2021;35:68–85. 34236624 10.1007/s12028-021-01227-y PMC8266715

[186] Chatelle C, Thibaut A, Gosseries O, Bruno MA, Demertzi A, Bernard C, et al. Changes in cerebral metabolism in patients with a minimally conscious state responding to zolpidem. Front Hum Neurosci. 2014;8:917. 25520636 10.3389/fnhum.2014.00917 PMC4251320

[187] Rodriguez-Rojas R, Machado C, Alvarez L, Carballo M, Estevez M, Perez-Nellar J, et al. Zolpidem induces paradoxical metabolic and vascular changes in a patient with PVS. Brain Inj. 2013;27:1320–9. 23924270 10.3109/02699052.2013.794961

[188] Du B, Shan A, Zhang Y, Zhong X, Chen D, Cai K. Zolpidem arouses patients in vegetative state after brain injury: quantitative evaluation and indications. Am J Med Sci. 2014;347:178–82. 23462249 10.1097/MAJ.0b013e318287c79c

[189] Kronberg G, Bridi M, Abel T, Bikson M, Parra LC. Direct current stimulation modulates LTP and LTD: activity dependence and dendritic effects. Brain Stimul. 2017;10:51–8. 28104085 10.1016/j.brs.2016.10.001 PMC5260488

[190] Kuo HI, Paulus W, Batsikadze G, Jamil A, Kuo MF, Nitsche MA. Acute and chronic effects of noradrenergic enhancement on transcranial direct current stimulation-induced neuroplasticity in humans. J Physiol. 2017;595:1305–14. 27925214 10.1113/JP273137 PMC5309376

[191] Cirillo G, Di Pino G, Capone F, Ranieri F, Florio L, Todisco V, et al. Neurobiological after-effects of non-invasive brain stimulation. Brain Stimul. 2017;10:1–18. 27931886 10.1016/j.brs.2016.11.009

[192] Fregni F, Boggio PS, Nitsche M, Bermpohl F, Antal A, Feredoes E, et al. Anodal transcranial direct current stimulation of prefrontal cortex enhances working memory. Exp Brain Res. 2005;166:23–30. 15999258 10.1007/s00221-005-2334-6

[193] Nelson JT, McKinley RA, Golob EJ, Warm JS, Parasuraman R. Enhancing vigilance in operators with prefrontal cortex transcranial direct current stimulation (tDCS). Neuroimage. 2014;85(Pt 3):909–17. 23235272 10.1016/j.neuroimage.2012.11.061

[194] Thibaut A, Bruno MA, Ledoux D, Demertzi A, Laureys S. tDCS in patients with disorders of consciousness: sham-controlled randomized double-blind study. Neurology. 2014;82:1112–8. 24574549 10.1212/WNL.0000000000000260

[195] Cincotta M, Giovannelli F, Chiaramonti R, Bianco G, Godone M, Battista D, et al. No effects of 20 Hz-rTMS of the primary motor cortex in vegetative state: a randomised, sham-controlled study. Cortex. 2015;71:368–76. 26301875 10.1016/j.cortex.2015.07.027

[196] Lutkenhoff ES, Chiang J, Tshibanda L, Kamau E, Kirsch M, Pickard JD, et al. Thalamic and extrathalamic mechanisms of consciousness after severe brain injury. Ann Neurol. 2015;78:68–76. 25893530 10.1002/ana.24423

[197] Schiff ND. Central thalamic deep brain stimulation to support anterior forebrain mesocircuit function in the severely injured brain. J Neural Transm (Vienna). 2016;123:797–806. 27113938 10.1007/s00702-016-1547-0

[198] Cain JA, Spivak NM, Coetzee JP, Crone JS, Johnson MA, Lutkenhoff ES, et al. Ultrasonic thalamic stimulation in chronic disorders of consciousness. Brain Stimul. 2021;14:301–3. 33465497 10.1016/j.brs.2021.01.008

[199] Monti MM, Schnakers C, Korb AS, Bystritsky A, Vespa PM. Non-invasive ultrasonic thalamic stimulation in disorders of consciousness after severe brain injury: a first-in-man report. Brain Stimul. 2016;9:940–1. 27567470 10.1016/j.brs.2016.07.008

[200] Zhou YF, Kang JW, Xiong Q, Feng Z, Dong XY. Transauricular vagus nerve stimulation for patients with disorders of consciousness: a randomized controlled clinical trial. Front Neurol. 2023;14:1133893. 36937511 10.3389/fneur.2023.1133893 PMC10017768

[201] Yifei W, Yi Y, Yu W, Jinling Z, Weihang Z, Shaoyuan LI, et al. Transcutaneous auricular vague nerve stimulation improved brain connection activity on patients of disorders of consciousness: a pilot study. J Tradit Chin Med. 2022;42:463–71. 35610018 10.19852/j.cnki.jtcm.2022.03.012 PMC9924658

[202] Kondziella D, Bender A, Diserens K, van Erp W, Estraneo A, Formisano R, et al. European Academy of Neurology guideline on the diagnosis of coma and other disorders of consciousness. Eur J Neurol. 2020;27:741–56. 32090418 10.1111/ene.14151

[203] Farisco M, Salles A. American and European Guidelines on disorders of consciousness: ethical challenges of implementation. J Head Trauma Rehabil. 2022;37:258–62. 35417436 10.1097/HTR.0000000000000776

[204] Graham M. Burying our mistakes: dealing with prognostic uncertainty after severe brain injury. Bioethics. 2020;34:612–9. 32124448 10.1111/bioe.12737 PMC7318633

[205] Souter MJ, Blissitt PA, Blosser S, Bonomo J, Greer D, Jichici D, et al. Recommendations for the critical care management of devastating brain injury: prognostication, psychosocial, and ethical management : a position statement for healthcare professionals from the Neurocritical Care Society. Neurocrit Care. 2015;23:4–13. 25894452 10.1007/s12028-015-0137-6

[206] Goostrey K, Muehlschlegel S. Prognostication and shared decision making in neurocritical care. BMJ. 2022;377: e060154. 35696329 10.1136/bmj-2021-060154

[207] Muehlschlegel S, Hwang DY, Flahive J, Quinn T, Lee C, Moskowitz J, et al. Goals-of-care decision aid for critically ill patients with TBI: development and feasibility testing. Neurology. 2020;95:e179–93. 32554766 10.1212/WNL.0000000000009770 PMC7455326

[208] Thibaut A, Di Perri C, Chatelle C, Bruno MA, Bahri MA, Wannez S, et al. Clinical response to tDCS depends on residual brain metabolism and grey matter integrity in patients with minimally conscious state. Brain Stimul. 2015;8:1116–23. 26471400 10.1016/j.brs.2015.07.024

[209] Piccione F, Cavinato M, Manganotti P, Formaggio E, Storti SF, Battistin L, et al. Behavioral and neurophysiological effects of repetitive transcranial magnetic stimulation on the minimally conscious state: a case study. Neurorehabil Neural Repair. 2011;25:98–102. 20647501 10.1177/1545968310369802

[210] Yu YT, Yang Y, Wang LB, Fang JL, Chen YY, He JH, et al. Transcutaneous auricular vagus nerve stimulation in disorders of consciousness monitored by fMRI: the first case report. Brain Stimul. 2017;10:328–30. 28017322 10.1016/j.brs.2016.12.004

[211] Corazzol M, Lio G, Lefevre A, Deiana G, Tell L, Andre-Obadia N, et al. Restoring consciousness with vagus nerve stimulation. Curr Biol. 2017;27:R994–6. 28950091 10.1016/j.cub.2017.07.060

[212] Timofeev I, Grenier F, Bazhenov M, Sejnowski TJ, Steriade M. Origin of slow cortical oscillations in deafferented cortical slabs. Cereb Cortex. 2000;10:1185–99. 11073868 10.1093/cercor/10.12.1185

[213] Silva LR, Amitai Y, Connors BW. Intrinsic oscillations of neocortex generated by layer 5 pyramidal neurons. Science. 1991;251:432–5. 1824881 10.1126/science.1824881

